# Supplementary material for: What motivates general practitioners to change practice behaviour? A qualitative study of audit and feedback group sessions in Dutch general practice
Source: BMJ Open. 2019 Jun 1;9(5):e025286. doi: 10.1136/bmjopen-2018-025286 (PMC6549704; doi:10.1136/bmjopen-2018-025286)
Supplement: Author's manuscript [file bmjopen-2018-025286.draft_revisions.pdf]

BMJ Open is committed to open peer review. As part of this commitment we make the peer review history of every article we publish publicly available.

When an article is published we post the peer reviewers' comments and the authors' responses online. We also post the versions of the paper that were used during peer review. These are the versions that the peer review comments apply to.

The versions of the paper that follow are the versions that were submitted during the peer review process. They are not the versions of record or the final published versions. They should not be cited or distributed as the published version of this manuscript.

BMJ Open is an open access journal and the full, final, typeset and author-corrected version of record of the manuscript is available on our site with no access controls, subscription charges or pay-per-view fees (http://bmjopen.bmj.com).

If you have any questions on BMJ Open's open peer review process please email info.bmjopen@bmj.com

BMJ Open

General Practitioners' motivation to change practice behavior: using mirror information in small-group discussions to improve care

Journal:	*BMJ Open*
Manuscript ID	bmjopen-2018-025286
Article Type:	Research
Date Submitted by the Author:	07-Jul-2018
Complete List of Authors:	van Braak, Marije; Erasmus MC, General Practice<br>Visser, Mechteld; Amsterdam Academic Medical Centre<br>Holtrop, Marije<br>Statius Muller, Ilona<br>Bont, Jettie; Amsterdam Academic Medical Centre<br>van Dijk, Nynke; Amsterdam Academic Medical Centre
Keywords:	MEDICAL EDUCATION & TRAINING, PRIMARY CARE, QUALITATIVE RESEARCH

SCHOLARONE™
Manuscripts

For peer review only

General Practitioners' motivation to change practice behavior: using mirror information in small-group discussions to improve care

Marije van Braak

Department of General Practice, Erasmus Medical Center

Postbus 2040, Wytemaweg 80, 3000 CA Rotterdam, the Netherlands

m.vanbraak@erasmusmc.nl

+31 10 7042518

Mechteld R. M. Visser, PhD

Department of General Practice, Academic Medical Center, Amsterdam, the Netherlands

Marije Holtrop, MD, GP

Amsterdam, the Netherlands

Ilona Statius Muller, MD, GP

Amsterdam, the Netherlands

Jettie Bont, MD, PhD, GP

Department of General Practice, Academic Medical Center, Amsterdam, the Netherlands

Nynke van Dijk, Professor, MD, PhD

Department of General Practice, Academic Medical Center, Amsterdam, the Netherlands

Word count: 4101

For peer review only

ABSTRACT

Background Dutch General Practitioners (GP) receive annual mirror information on their professional performance in terms of consultation, intervention, prescription and referral rates.

Objectives Adopting a motivational perspective, we investigate 1) what factors motivate GPs to change their practice behavior to improve care based on mirror information, and 2) to what extent small-group discussions of mirror information contribute to GPs' practice behavior change.

Methods Four focus groups, comprising a total of 39 participating local GPs (i.e., with practices in/close to Amsterdam), discussed and compared mirror information of their practices.

Results GPs' self-reported motivation to change was generally low or absent. GPs judged it *impossible* to change practice behaviors attributed to other professionals, they *refused* to change particular practices due to their convictions regarding good practice, and they regarded change as simply *undesirable* because of the multi-interpretable and outdated nature of the mirror information. However, participating GPs evaluated the peer discussion of mirror information positively. They felt provoked to critically appraise mirror information and experienced a need for behavioral change during the interactive discussion and comparison with others.

Conclusions For mirror information to potentially motivate GPs to change their current practice, it should be up-to-date, reliable, specific, and concern practitioners' individual behavior. Peer interaction can positively contribute to explorations of alternative practices and avenues for improvement, and local or regional peer meetings would be beneficial in facilitating reflection and discussion. An important avenue for future studies is to explore the contribution of mirror information and small-group discussion to *actual* practice change.

For peer review only

STRENGTHS AND LIMITATIONS OF THIS STUDY

- Focus group discussions on personal and comparative mirror information allowed us to tap into real-time reflective processes and the diversity of attributions and factors that impact GPs' motivation to change.
- Qualitative analysis of recorded interaction between GPs allowed for detailed insight into the value of peer interaction in discussions on practice change.
- Voluntary GP participation may have resulted in a sample of participants with a special interest in mirror information and behavior change.
- The study focused only on effects of mirror information and group discussion on intended, not actual, practice change.

For peer review only

INTRODUCTION

Affirming the Hippocratic oath, all doctors, including general practitioners, treat patients to the best of their ability. Despite this shared pursuit, however, care practices differ substantially between general practitioners.(1-3) Part of this diversity is induced by external practice or population factors, such as practice size.(2) Additionally, general practitioners' personal choices, preferences, knowledge, and capabilities can induce between-practice variation. This variation is often related to differences in personal decisions based on evidence, costs and patient satisfaction,(4) but sometimes is not intended and may lead to lower patient care quality.

For general practitioners (GPs) to develop their professional practice, they should be aware of this unintended variation. Reflection on between-practice variation can lead to adjustments in professional care behavior, eventually improving the quality of patient care.(5) Feedback on GPs' performance can effectively improve professional practice, albeit under certain optimally-designed conditions and in the right context.(6) Several factors influencing the effectiveness of feedback have been researched. These include the level of feedback detail,(7) its timing, (8, 9) and the interactivity of the feedback-giving process.(4, 5, 9, 10)

In addition to these situational conditions, a particularly significant factor influencing the effectiveness of feedback on performance is GPs' motivation and willingness to change.(11-13) Motivation is essential to learning and change processes.(14) In a study on physicians' prescription behavior, Wakefield et al.(15) found that physicians who expressed a commitment to change following participation in a continuing medical education program using interactive small groups were significantly more likely to change their targeted prescription practices in the following half year. Contradictory evidence, however, has also been reported.(16, 17) Palmer(16) assessed the motivation of health professionals to change their practices and found that they improved on tasks for which they reported to have *limited* motivation. This counterintuitive finding can be explained by the significant *system* changes (in addition to individual changes) required to change practices for which professionals' motivation was high. Thus, the relation between motivation and care practice improvement may be less straightforward than originally anticipated.

For peer review only

The reported variability in the effect of motivation on actual care practice change can be explained by attributional processes that impact an individual's motivation to change.(18) For example, GPs might attribute personal practice variation to 'the system', particular patients, or fellow healthcare professionals (i.e., hold others responsible for practice variation). In such a case, the GPs are not likely to be inclined to change their own professional practice behavior. Thus, GPs' attributions are significant in processes of general practice change.

In the current study, we qualitatively investigated willingness to change professional behavior based on feedback in the form of *mirror information* and *peer interaction*. Generation of this study's mirror information was initiated by the Dutch movement 'Optimale zorg-Dappere dokters' [Optimal care-Daring doctors], with the aim of encouraging GPs' self-reflection and optimizing care. Group-based critical self-evaluation is not included in contemporary quality policies of the medical profession, but might be beneficial to stimulate self-reflection for optimal care. Evaluating collaborative discussions of mirror information, then, is a crucial step in establishing an evidence base for effective interventions that stimulate GPs to become optimal care providers. In our study, we adopted a motivational perspective and focused on GPs' personal and environmental attributions of current practice reality. With the aim of developing and evaluating an intervention for GPs' professional development, the research was guided by the following research questions:

1. What factors motivate GPs to change their practice behavior to improve care based on mirror information?
2. To what extent do small-group discussions of mirror information contribute to GPs' practice behavior change?

THEORETICAL FRAMEWORK

The role of attributions in motivation and behavior change is described in Weiner's Attribution Theory,(19, 20) which indicates that humans "have a tacit goal of understanding and mastering themselves and their environment" and "establish cause-effect relationships for events in their lives".(18) Occurring events lead to attribution, a process of (often subconsciously) seeking an explanation by hypothesizing perceived personal and environmental causes (e.g., ability, effort, luck, task difficulty, mood, health, other people, etc.). These causes can be organized along three causal

For peer review only

dimensions: locus (i.e., internal or external to the individual), stability (i.e., stable, fixed or unstable, likely to change), and controllability (i.e., within or outside the individual's control). Based on the specific combination of values in each dimension, occurring events are interpreted as psychologically meaningful responses. As such, the individual's *interpretation* of a particular event determines their response to that event. Typically, bad luck is interpreted as external, unstable and uncontrollable – invoking no or possibly only a passive reaction from the individual; personal effort is internal, changeable, and controllable – and can thus be influenced by an individual's actions; and innate skill is internal, largely fixed, and uncontrollable – making it an unlikely subject of change.(18)

METHODS

Participants

We conducted four focus group discussions(21, 22) with 39 GPs from four regional groups in/close to Amsterdam. Focus groups comprised 7-10 GPs from the same region, ranging in age from 30 to 65 years of age (the majority being over 50 years of age). Approximately 75 percent of participating GPs work in a practice together with a partner, with the remainder working alone or in a group practice.

Since specialist care in the Netherlands is only accessible upon referral by a GP, Dutch general practice plays an important role in the Dutch health care system.(23) All Dutch citizens are registered at a GP-practice in their regional area. Patients visit their GP when faced with a medical problem (except in life-threatening situations). The GP collects and evaluates all relevant medical information. Consequent treatment decisions and referrals to a medical specialist are taken together with the patient. Costs of care are covered by healthcare insurances, which are compulsory for people who live or work in the Netherlands and which include at least basic health care.

The Medical Ethics Review Committee of the Amsterdam Academic Medical Centre confirmed that the Medical Research Involving Human Subjects Act does not apply to this study and that an official approval of this study by the committee was not required (reference number W18_200 # 18.241).

Procedure

General practitioners from the four regional groups were approached and informed about the research purpose and participation practicalities (via an information letter). Participating GPs signed informed

For peer review only

consent prior to the discussions, held between June 2016 and March 2017 at a participating GP's practice, which lasted approximately 1.5 hours. Sessions were audio-taped for transcription purposes.

Each group discussion started with a short introduction from the moderator (ND or MV), detailing the aim of the meeting. Consequently, participants were encouraged to ask questions or share striking aspects of their individual mirror information (data from 2012-2014). Mirror information is based on declarations of health care costs to insurance companies, gathered and provided to GPs by Vektis, a Dutch center for information and standardization of health insurance. It provides data on a practice's professional performance in terms of practice population, consultations, interventions, prescription, and referral rates compared with a standardized Dutch practice, corrected for age, gender, social-economic status and disease severity of the population. Subsequent group discussion focused on comparative mirror information which was constructed by the research group prior to each meeting, based on a standardized analysis of general practitioners' mirror information (sent by the practitioners to the research group upon confirmation of participation). Comparison information was presented via diagrams; actual practice mirror information was only available to general practitioners of that practice, but was shared with other general practitioners if relevant to the discussion or upon practitioners' own initiation. The focus group discussion concluded with an exploration of potential issues relevant for a more in-depth future discussion. Two follow-up sessions were organized to discuss the agreed-upon issues (not reported on in this paper).

Data

The four focus group interactions were transcribed verbatim and anonymized (geographical and personal names were deleted). Fragments unintelligible due to simultaneous speech or laughing were transcribed as [unintelligible] and included a timestamp to facilitate fragment location at a later moment of analysis if needed.

Analysis

Analysis of the focus group discussions was based on the transcripts, and audiotapes were consulted to improve interpretation where necessary. Transcripts were analyzed using principles of Grounded Theory.(24, 25) Although originally aimed at *theory* development, Grounded Theory in medical education research has also been used to achieve "adequate *understanding* for specified contexts and

For peer review only

purposes".(26) (27) We employed Grounded Theory to achieve a thorough understanding of GPs' motivation to change their care practices and factors influencing this motivation.

Following the principles of Grounded Theory, transcripts were analyzed step-by-step using MAXQDA software.(28) First, we derived from motivation and attribution theory a number of concepts relevant to our motivational perspective on practice change. These 'constructed codes' formed the initial framework for data coding.(25) The unit of analysis was determined to be a fragment of speech by one or more participants concerning one particular, demarcated topic.

Second, MB coded a first transcript, during which constructed codes were supplemented with open in vivo codes derived inductively from the data (e.g., *gaining information* or *evaluation*). A codebook including code descriptions and coding rules was developed to support the coding process. Two authors (MB and ND) discussed the coding of this first transcript to ensure coding reliability. Several codes were adapted slightly and some were merged for code reduction and reorganization (axial coding).(25)

In the third step, MB and ND independently coded a second focus group transcript using the codes resulting from step two, adding additional open codes where needed. The authors' codings of each fragment were compared and discussed in detail until agreement was reached, again resulting in several adjustments to the codes and consequently to previously coded transcripts.

Fourth, MB used the developed coding tree to code transcripts of focus groups three and four. Reliability of coding was assured by discussing with ND those fragments that could not unambiguously be assigned a code until agreement was reached. Although the content of focus groups three and four differed from the content of focus groups one and two in several respects (e.g., topics discussed and distribution of time devoted to discussing actual mirror information or behavioral change based on mirror information), the existing codes sufficed to cover the content. Data saturation was thus reached.

The final step in Grounded Theory is to synthesize data by establishing relationships between code categories to make sense of what is happening in the field.(25) We reassembled the data to formulate a core category or central understanding by identifying the most important code categories,

For peer review only

concepts, or themes, looking for connections between them, and formulating the significant message conveyed by participants during the analyzed discussions.

RESULTS

Generally, discussions of a particular aspect of the mirror information commenced with resolving potential difficulties in reading or interpreting that particular piece of information. Subsequently, GPs construed an understanding of the information, focusing on probable explanations for deviations from average or differences between practices and formulating motivations for change. GPs often used attributions to explain hesitations to change an aspect of practice behavior, i.e., explaining causes of their presented care behavior. In what follows, we present the motivating and demotivating factors for care practice behavior based on mirror information and contributions from small-group discussions, and show how the attributions influenced GPs' choices to change their current practice. Finally, we discuss GPs' ideas for how to increase motivations for change.

Motivations to change care practice behavior

Participating GPs mentioned several motivations for changing their current practice behavior, many of which arose from increased *awareness* about aspects of their current care practice. A heightened awareness of one's own practices (as reported in the mirror information), sometimes led to the realization that actual care practices differed extensively from perceived care practices or from the norm. This awareness frequently induced GPs to express intentions to further reflect on or take additional steps towards practice change (e.g., Box 1).

Box 1 *Focus group O*

1	GP A	Well, what I do want to do is check all our cyriax cases to see what the indications were. That is
2		quite a job and it would be very nice if we could receive that mirror information. But this
3		finding has already provoked me to look that up in my own Electronic Health Record to see how
4		many have been done.
5	CHA	Yes.
6	GP A	Then we would really have something to talk about, I think.

As this excerpt reveals, being confronted with specific mirror information about one's own practice (e.g., the finding that the number of cyriax operations deviates considerably from the norm, line 3) can lead to undertaking specific steps to understand and potentially adapt one's care practices.

For peer review only

At times, discussing care practices led to an explicitly formulated realization that particular practices had to be adjusted for reasons proposed during the discussion. As indicated in Box 2, one participating GP realized that his hesitation to plan double consults (20 minutes for patients with multiple complaints) instead of the common 10 minute-consult (the preferred Dutch general practice choice) was unjustified.

Box 2 *Focus group O*

1	GP A	This is such an eye opener for me, you know? I constantly feel guilty when I plan twenty-
2		minute consults twice a day, because then my assistants won't be able to schedule
3		enough patients – that's what I think. But I can only do that!
4	GP B	But darling, we are manning just slightly more patients with twice as many practitioners as you
5		have.
6	GP A	Yes, that's true.
7	GP B	You know, I mean - that is how it is, really.
8	GP C	Yes, so you need an extra doctor, and more double consults.
9	GP A	And a room.
10	GP C	Yes, and if we charge for the double consults as well, then an extra doctor does not cost a thing.
11	GP D	Indeed, then you recover the expenses easily.

Other GPs participating in this interaction are used to planning double consults and do not experience the time issues that GP A mentions in lines 1-2. The reason behind the differences in the number of double consults planned, GP B poses, is probably understaffing (line 4). Following this suggestion, GP C and GP D formulate an explicit need for change (lines 5-6), and potential objections to the proposed change are warded off in lines 7-8. This excerpt shows the benefits of discussing one's mirror information with fellow GPs.

During the focus group discussions, several GPs indicated their motivation to change current care behavior by comparing mirror information, asking critical questions and verifying reasons/causes behind the practices represented by the figures. However, comparison is not always perceived to be informative, as revealed in Box 3.

Box 3 *Focus group OD*

1	GP A	I think that is interesting, when we see - when I see that I deviate from the national average, and
2		we all deviate, then you think: what do we do about that?
3	GP B	Sure.
4	GP A	If only mine deviates, then I think: well, I have to do something about that.

For peer review only

Apparently, the need (and motivation) to change practices is more pressing if only one GP's practice deviates from the norm compared to deviation common to all participating GPs. Common deviations are often attributed to demographical or geographical characteristics. Comparing each other's mirror information thus functions as a filter, isolating idiosyncratic practice-related variation from region-bound practice variation.

Despite regularly motivating GPs to change care practices, group discussions sometimes merely initiated sharing of motivations behind and reflections on practice behavior without triggering practice change. This type of motivated sharing of best practices occurred quite frequently throughout the group discussions.

In general, group discussion is perceived to facilitate interpretation of mirror information and evaluations of the need for practice change. Most of the practice behavior that GPs are motivated to change is their own, or that within their own influence sphere. GPs can control these behaviors, thus making motivation to change more likely compared to behavior outside of GPs' control. As clarified in the next section, however, much of the mirror information is about practices that are outside of GPs' control.

Motivations to *not* change care practice behavior

Typically, GPs expressed *no* intentions to change their practice; moreover, mentions of motivations to *not* change care practice behavior are relatively common. Three categories of motivations to not change practices were attested, i.e., because it was *impossible* to do so, because GPs were *unwilling* to do so, or because GPs judged it *undesirable* to change behavior based on the provided mirror information.

Impossible to change

Confronted with specific figures about referral or prescription rates in the mirror information, GPs proposed several reasons for the impossibility of changing practices. Each of these is an attribution of the information to someone or something external to the individual GP.

First, some figures would not only represent the GP's own behavior, but also others' behavior (i.e., be the result of other people's actions). For example, several figures include prescriptions by the GP him/herself, but also specialist-prescriptions – prescription behavior which cannot easily be

For peer review only

influenced by the GP (although GPs suggested that they talk to a specialist to discuss deviant figures or refuse particular referrals). Similarly, norm deviations are sometimes attributed to the presence of a GP-in-training at the time of measurement (e.g., resulting in more applications for radiology or lab diagnostics), the location of the practice (e.g., resulting in fewer home visits if located close to an old-age home or in the care center), treatments by non-GP health professionals (e.g., in-hospital treatment) or other GPs (e.g., if other GPs declare particular treatments erroneously while a participating GP does it correctly, this GP's declaration rates will deviate from the norm despite his declaration practices being 'correct'), or individual patients (e.g., who might prefer a particular treatment over others).

Non-human, external factors are also mentioned as reasons for the impossibility of practice change. Examples are regulations laid down in codes of conduct and time issues (see Box 4).

Box 4 *Focus group OD*

1	GP A	I do tell them quite often to come - make a new appointment, but I do have too few consults
2		already as well.
3	GP B	Oh so that doesn't help either.
4	GP A	So that doesn't help either.
5	GP C	We cannot even schedule more consults.
6	GP D	Full is full.
7	GP C	Yes, only if you want to continue working through the evening.

Occasionally, GPs indicated that a particular care practice is impossible without explaining why. These practices just "are the way they are". Despite being potentially problematic, change is very difficult to initiate. The quote below exemplifies this in the context of long waiting times for mental care in the participating GPs' geographical area.

Box 5 *Focus group P*

1	GP A	There has always been a shortage of psychiatric care in [name city].
2	GP B	That's psychiatric care, but basic - just basic mental health care has of course had a surplus of
3		primary care psychologists.
4	GP C	I don't know, I still have waiting times quite often.
5	GP D	There have been long waiting times for sure.
6	CHA	So you say that actually it is the desired variation, it is variation, we have much of that, but it is
7		like it is.
8	GP E	No, it is- I acknowledge it now, that it isn't easily solved.

Although it is suggested that the issue at hand (variation in length of waiting time) is not problematic, the main point illustrated in this quote is that some deviations from the norm are the result of issues far

For peer review only

beyond the behavior of the GPs themselves, reducing motivation to change their own practices in the first place.

Unwilling to change

At times, GPs *can* change a certain practice, but are *not willing to*. GPs were convinced sometimes that different practices may be right (or at least acceptable), despite resulting in a deviation from the norm. Quite commonly, GPs employed logical reasoning to explain their unwillingness to change particular practice behavior. In the following example, the unexpectedly high level of IUDs reported for one of the general practices is attributed to the gynecological background of a GP employed at that practice.

Box 6 *Focus group O*

1	CHA	Minor surgery.
2	GP A	So these are the young women with these IUDs.
3	CHA	For you, those are the IUDs indeed, those are quite high in your figures.
4	GP B	Yes.
5	CHA	I haven't seen that with any of the other practices.
6	GP B	No.
7	CHA	So many...
8	GP B	That is Dr. [name], he has a gynecological background.

Apparently, the doctor's background is sufficient ground for not changing current practice; in fact, this background is used as a rational explanation and validation of the attested variation. Participating GPs did not interpret this deviation from the norm as an indication of a need for change, neither at the Dr.'s nor at the others' practices.

A third reason mentioned for not wanting to change practice behavior is the irrelevance of certain differences between current and norm practice. Minimal deviations from norm referral, prescription, and treatment rates are judged irrelevant to future practice change. Potential changes that would affect only a limited number of patients are viewed as trivial, as pointed out by GP A:

Box 7 *Focus group P*

1	GP A	There is one other thing that I appreciate about these figures, sometimes deviations are
2		enormous but then it's only about 10 patients, one isn't going to change policy on that.

Undesirable to change

For peer review only

A third recurrent theme in GPs motivations to not change behavior is the nature of the provided mirror information. According to GPs, it is undesirable to change certain current practices based on mirror information presented and discussed during the group discussions. The mirror information is commonly considered unreliable or invalid. At one point, a GP explicitly suggests that a computer error has been made, and mirror information is regularly questioned on the basis of one's own experience or feeling about the frequency of applying a particular practice. Suspicion of unreliability of the mirror information induces insecurity about possible future actions; one GP said: "it doesn't match with how I feel about it (…), so I don't really know what I should or could do with that". According to another GP, only reliable figures that resemble the GP's own behavior can rightfully trigger change.

Several GPs stated that mirror information, besides being unreliable in some respects, is also meaningless to some extent. The mirror information figures provide information about frequencies and percentages, but the reader cannot tell from them what comprises the figures. It remains unclear why increases and decreases in prescriptions, referrals, and treatments occur and to whom (or what) these changes can be attributed (see Box 8).

Box 8 *Focus group V*

GP A	But then one soon asks oneself: "How did they get to these figures? How is all of this calculated, if the difference is so large, we haven't started working completely differently a year later". So there is something there. That does give a lot of interesting information (…). It always comes down to: What are your norms, why - how are things actually counted? Yes, that's when things get terribly difficult. That says a lot about the reliability.
GP B	I would say they have started to count in a different way.

Thus, based on perceived limited reliability and clarity, potential changes of care practices are commonly judged undesirable – or are not even considered at all.

Increasing GPs' motivation to change

For mirror information to facilitate and motivate change, GPs indicate that it should be more specific, recent, and adaptive. Regarding specificity, GPs appreciate mirror information specific for each GP in the case of a dual or multi-practice. GPs would benefit from broad themes being split up into smaller subthemes (e.g., ECGs for specific problems instead of one figure for all ECGs made). The following quote illustrates the general perception of the felt need for specificity.

For peer review only

Box 9 *Focus group P*

1	GP A	Those figures are very broad and big, really, so I find it difficult to - if one gets back something
2		small from your figures, something that you can improve on easily and if one gets back the
3		figures again after half a year, then I would be more likely to show behavior change.

In addition to a desire for specificity, GPs express a wish for more recent information. Short term feedback is agreed upon as being critical to the effectiveness of mirror information as facilitator of change. GPs do not feel the urge to 'learn' from figures that represent their behavior registered three or more years ago.

Finally, GPs call for more adaptive feedback: mirror information that would give insight into specific prescriptions or patient information in the case of, for example, extreme prescription rates or costs (as one GP with high costs wondered: "Is that because of that Augmentin for that cat bite?"), and would be available on request. Having the option to link prescriptions, referrals, and costs to specific patients would make the system more useful to GPs.

DISCUSSION

The present investigation into GPs' motivations to change their current practice behavior based on mirror information about consultation, intervention, prescription, and referral rates reveals three main conclusions. First, despite GPs' motivation to change based on presented mirror information generally being low or absent, mirror information does lead to a heightened awareness of one's own and alternative practice behavior. At times, that awareness propels GPs to the next step towards change.(12) The extent to which practices pertain to GPs' own, individual, directly-controllable behavior is a strong factor in inducing GPs' expressions of change intention in individual conduct. Current practices that GPs *do not* intend to change generally involve behaviors attributed to others. As such, these behaviors are an unlikely subject for behavior change.(18)

Second, change is conditional on GPs' feeling of urgency. The presented mirror information is generally not well-suited to induce the urgency needed to prompt change. Mirror information ought to be *up-to-date*, *reliable*, *specific*, and concern data on practitioners' *individual* behavior to potentially effectuate change. This finding endorses the cardinal importance of high quality mirror information discussed in investigations of mirror information effectiveness in contexts other than General

For peer review only

Practice.(7-9) Simultaneously, and more practically, these results indicate that significant adjustment of contemporary Dutch mirror information is required to assure prompt and profitable use for GP professionalization.

Third, our findings point to the key role of collectively discussing mirror information. Other than the mirror information itself, small-group peer discussion frequently heightens the perceived urgency for action. As indicated by Trietsch et al., social influence and norms affect participants' reflective behavior and corresponding intentions to change current practice during peer interaction.(29) Contrasting with Ivers et al.'s conclusion that there is very limited evidence for peer-comparison mirror information being either more or less effective than individual performance information,(6) our data show that peer comparison in general and group processes in particular stimulate critical appraisal of the mirror information and the need for behavioral change. Peer comparison provides an interpretative framework for individual practice data and peer interactivity provides ample opportunities to explore alternative practices and promising avenues for improvement.(5, 10)

Practically, our findings imply a need for annual provision of recent practitioner-related mirror information. For GPs to be informed about particular (e.g., deviant) treatments, referrals, and interventions in greater detail, mirror information needs to be adaptive, i.e., provide the possibility to investigate closer exactly those aspects that a particular GP would like to learn more about. Optionally, already accessible practice-related information (e.g., in the Electronic Health Record) can be employed as a starting point for an informative, easily accessible, and adaptively employable application serving GP professionalization. Additionally, frequent meetings with GPs practicing in the same local or regional area to facilitate group reflection and discussion would be very valuable.

The present investigation of GPs' motivations to change or not to change current practices thus offers a detailed understanding of GPs' change considerations. By conducting focus group discussion based on personal and comparative mirror information, we were able to tap into real-time communicated reflective processes and sketch a comprehensive image of the diversity of attributions and factors impacting GPs' motivation to change. The actual effects of mirror information and group discussion on practice change, however, have not been measured. Given the inconclusive evidence on

For peer review only

the extent to which intentions to change practice are predictive of actual practice change,(15, 30) future work should explore the correspondence between intended and actual change. More specifically, determining the effects of mirror information on diverse aspects of care quality (patient health, patient satisfaction, costs) – both in the short and the long term – is crucial to determining promising directions for GP professionalization.

Despite the use of a specific type of mirror information, which, as outlined above, has its own particular shortcomings and demands cautious interpretations as far as results relating to features of this specific type of mirror information are concerned, the current study's focus on mirror information use in daily practice has certainly enriched our understanding of the complexities of those processes that jointly foster GPs' professionalization: individual reflection and critical discussion.

For peer review only

Contributors

All authors contributed substantially to the conception, design, or execution of the reported study. MH and ISM, in collaboration with ND, took initiative for the study. ND, MV, MH and ISM designed the study and collected the data. JB participated in the interpretation of the data; MB, ND and MV participated in data analysis. MB was responsible for the write-up of the study. All authors critically revised its content and provided final approval of the version to be published.

Funding

This research received no specific grant from any funding agency in the public, commercial or not-for-profit sectors.

Competing interests

None declared.

Data sharing

The data for this paper may be obtained from the authors upon request.

Formatted: Dutch (Netherlands)

For peer review only

REFERENCES

1. Koefoed MM, Søndergaard J, Christensen Rd, et al. General practice variation in spirometry testing among patients receiving first-time prescriptions for medication targeting obstructive lung disease in Denmark: a population-based observational study. *BMC Fam Pract* 2013;14:113.

2. Millett C, Car J, Eldred D, et al. Diabetes prevalence, process of care and outcomes in relation to practice size, caseload and deprivation: national cross-sectional study in primary care. *J R Soc Med* 2007;100(6):275-83.

3. Nielen MMJ, Schellevis FG, Verheij RA. Inter-practice variation in diagnosing hypertension and diabetes mellitus: a cross-sectional study in general practice. *BMC Fam Pract* 2009;10:6.

4. Verstappen WM, van der Weijden T, Sijbrandij J, et al. Effect of a practice-based strategy on test ordering performance of primary care physicians: A randomized trial. *JAMA* 2003;289(18):2407-12.

5. Cervero RM, Gaines JK. The Impact of CME on Physician Performance and Patient Health Outcomes: An Updated Synthesis of Systematic Reviews. *J Contin Educ Health Prof* 2015;35(2):131-8.

6. Ivers N, Jamtvedt G, Flottorp S, et al. Audit and feedback: effects on professional practice and health care outcomes. *Cochrane Database Syst Rev* 2012(6):1-227.

7. O'Connell DL, Henry D, Tomlins R. Randomised controlled trial of effect of feedback on general practitioners' prescribing in Australia. *BMJ* 1999;318(7182):507-11.

8. Mugford M, Banfield P, O'Hanlon M. Effects of feedback of information on clinical practice: A review. *BMJ* 1991;303(6799):398-402.

9. Axt-Adam P, van der Wouden JC, van der Does E. Influencing behavior of physicians ordering laboratory tests: A literature study. *Med Care* 1993;31(9):784-94.

10. Verstappen WHJM, van der Weijden T, Dubois WI, et al. Improving test ordering in primary care: The added value of a small-group quality improvement strategy compared with classic feedback only. *Ann Fam Med* 2004;2(6):569-75.

11. Fox R, Mazmanian P, Putnam RW. Changing and learning in the lives of physicians. New York: Praeger 1989.

For peer review only

12. Grol R, Wensing M. What drives change? Barriers to and incentives for achieving evidence-based practice. *Med J Aus* 2004;180(6 Suppl):S57-60.

13. Kanouse DE, Jacoby I. When does information change practitioners' behavior? *Int J Technol Assess Health Care* 1988;4(1):27-33.

14. Kusurkar RA, Artino AR, Ten Cate TJ. Motivation and learning. In: Bhuiyan PS, Rege NN, Supe A, eds. The art of teaching medical students. New Delhi: Reed Elsevier India Pvt. Ltd. 2015:62-76.

15. Wakefield J, Herbert CP, Maclure M, et al. Commitment to change statements can predict actual change in practice. *J Contin Educ Health Prof* 2003;23(2):81-92.

16. Palmer RH, Louis TA, Hsu L-N, et al. A randomized controlled trial of quality assurance in sixteen ambulatory care practices. *Med Care* 1985;23(6):751-70.

17. Foy R, MacLennan G, Grimshaw J, et al. Attributes of clinical recommendations that influence change in practice following audit and feedback. *J Clin Epidemiol* 2002;55(7):717-22.

18. Cook DA, Artino AR. Motivation to learn: an overview of contemporary theories. *Med Educ* 2016;50(10):997-1014.

19. Weiner B. An Attributional Theory of Achievement Motivation and Emotion. *Psychol Rev* 1985;92(4):548-73.

20. Weiner B. Intrapersonal and Interpersonal Theories of Motivation from an Attributional Perspective. *Educ Psychol Rev* 2000;12(1):1-14.

21. Krueger RA, Casey MA. Focus groups. A practical guide for applied research. Thousand Oaks, CA: SAGE Publications, Inc.; 2000.

22. Tong A, Sainsbury P, Craig J. Consolidated criteria for reporting qualitative research (COREQ): a 32-item checklist for interviews and focus groups. *Int J Qual Health Care* 2007;19(6):349-57.

23. Vektis. Zorgprisma Publiek: Huisartsenzorg. 2017. https://www.zorgprismapubliek.nl/informatie-over/huisartsenzorg/huisartsenzorg/ (accessed June 2017).

24. Glaser BG, Strauss AL. The discovery of Grounded Theory: strategies for qualitative research. Chicago, IL: Aldine; 1967.

25. Boeije H. Analysis in qualitative research. London: SAGE Publications Ltd 2010.

For peer review only

26. Watling CJ, Lingard L. Grounded theory in medical education research: AMEE guide no. 70. *Med Teach* 2012;34(10):850-61.

27. Bryant A. Re-grounding grounded theory. *Journal of Information Technology Theory and Application* 2002;4(1):25-42.

28. MAXQDA [Computer software for qualitative data analysis]. Berlin: VERBI Software Consult Sozialforschung GmbH 1989-2017.

29. Trietsch J, van der Weijden T, Verstappen W, et al. A cluster randomized controlled trial aimed at implementation of local quality improvement collaboratives to improve prescribing and test ordering performance of general practitioners: Study Protocol. *Implement Sci* 2009;4:6.

30. Overton GK, McCalister P, Kelly D, et al. Practice-based small group learning: How health professionals view their intention to change and the process of implementing change in practice. *Med Teach* 2009;31(11):e514-e20.

For peer review only

BMJ Open

What motivates General Practitioners to change practice behavior? A qualitative study of audit and feedback group sessions in Dutch General Practice

Journal:	*BMJ Open*
Manuscript ID	bmjopen-2018-025286.R1
Article Type:	Research
Date Submitted by the Author:	22-Nov-2018

Complete List of Authors:	van Braak, Marije; Erasmus Medical Centre, General Practice<br>Visser, Mechteld; Amsterdam University Medical Centre, General Practice<br>Holtrop, Marije; General Practitioners Holtrop and Sieben<br>Statius Muller, Ilona; Ubbens and Statius Muller General Practitioners<br>Bont, Jettie; Amsterdam University Medical Centre, General Practice<br>van Dijk, Nynke; Amsterdam University Medical Centre, General Practice

**Primary Subject Heading**:	General practice / Family practice
Secondary Subject Heading:	Medical education and training, Qualitative research
Keywords:	MEDICAL EDUCATION & TRAINING, PRIMARY CARE, QUALITATIVE RESEARCH

SCHOLARONE™ Manuscripts

What motivates General Practitioners to change practice behavior? A qualitative study of audit and feedback group sessions in Dutch General Practice

Marije van Braak, MSc

Department of General Practice, Erasmus Medical Centre

Postbus 2040, Dr. Molewaterplein 40, 3015 GD Rotterdam, the Netherlands

m.vanbraak@erasmusmc.nl

+31 10 7042518

Mechteld R. M. Visser, PhD

Department of General Practice, Amsterdam University Medical Centre, Amsterdam, the Netherlands

Marije Holtrop, MD, GP

General Practitioners Holtrop and Sieben

Amsterdam, the Netherlands

Ilona Statius Muller, MD, GP

Ubbens and Statius Muller General Practitioners

Amsterdam, the Netherlands

Jettie Bont, MD, PhD, GP

Department of General Practice, Amsterdam University Medical Centre, Amsterdam, the Netherlands

Nynke van Dijk, Professor, MD, PhD

Department of General Practice, Amsterdam University Medical Centre, Amsterdam, the Netherlands

Word count: 4126

For peer review only

ABSTRACT

Objectives Adopting an attributional perspective, the current article investigates how audit and feedback group sessions contribute to GPs' motivation to change their practice behavior to improve care. We focus on the contributions of the audit and feedback itself (content) and the group discussion (process).

Methods Four focus groups, comprising a total of 39 participating Dutch GPs, discussed and compared audit and feedback of their practices. The focus groups were analyzed thematically.

Results Audit and feedback contributed to GPs' motivation to change in two ways: by raising awareness about aspects of their current care practice and by providing indications of the possible impact of change. For these contributions to play out, the audit and feedback should be reliable and valid, specific, recent and recurrent, and concern GPs' own practices or practices within their own influence sphere. Care behavior attributed to external, uncontrollable, or unstable causes would not induce change. The added value of the group is twofold as well: group discussion contributed to GPs' motivation to change by providing a frame of reference and by affording insights that participants would not have been able to achieve on their own.

Conclusions In audit and feedback group sessions, both audit and feedback information and group discussion can valuably contribute to GPs' motivation to change care practice behavior. Peer interaction can positively contribute to explore alternative practices and avenues for improvement. Local or regional peer meetings would be beneficial in facilitating reflection and discussion. An important avenue for future studies is to explore the contribution of audit and feedback and small-group discussion to *actual* practice change.

For peer review only

STRENGTHS AND LIMITATIONS OF THIS STUDY

- Framed within Attribution Theory, the study provides a novel perspective on audit and feedback.
- Focus group discussions on personal and comparative audit and feedback allowed us to tap into real-time reflective processes.
- Qualitative analysis of recorded interaction between GPs allowed for detailed insight into the value of peer interaction in discussions on practice change.
- Voluntary GP participation may have resulted in a sample of participants with a special interest in audit and feedback and behavior change.
- The study focused only on effects of audit and feedback and group discussion on intended, not actual, practice change.

For peer review only

INTRODUCTION

In taking the Hippocratic oath, general practitioners – and other doctors as well – express their intention to treat patients to the best of their ability. Yet, care practices of general practitioners show substantial unintended variation.(1-3) Part of this diversity is induced by external practice or population factors, such as practice size.(2) Individual factors also play a role: general practitioners' knowledge, skills, experience, interests and preferences can induce between-practice variation. This variation might be related to differences in clinical judgment based on considerations of evidence, clinical experience and patient preferences,(4) but sometimes is not intended and may lead to lower patient care quality.

For general practitioners (GPs) to develop their professional practice, they should be aware of this unintended variation. Reflection on between-practice variation can lead to adjustments in professional care behavior, eventually improving the quality of patient care.(5) Audit and feedback on GPs' performance can effectively improve professional practice, albeit under certain optimally-designed conditions and in the right context.(6) Several factors influencing the effectiveness of feedback have been researched. These include the level of feedback detail,(7) its timing, (8, 9) and the interactivity of the feedback-giving process.(4, 5, 9, 10) Based on expert interviews, systematic reviews, and experience, Brehaut and colleagues suggest that practice feedback interventions can be optimized by, amongst others, linking it to established goals, providing feedback in more than one way, minimizing extraneous cognitive load for feedback recipients, increasing the credibility of the data and preventing defensive reactions to feedback.(11) Additionally, recent research on group audit and feedback points at the added value of socially constructed learning activities in audit and feedback group sessions.(12)

The theoretical framing of audit and feedback research is diverse, ranging from feedback theories (e.g., Feedback Intervention Theory, (13)) to psychological theories (e.g. Self Affirmation Theory, (14)) to implementation theories (e.g. Consolidated framework for implementation research, (15)) to learning theories (e.g. Social Learning Theory, (16)).(17) In our study, we explicitly focus on GPs' *motivation* to change. This perspective on audit and feedback has not been used before. Yet, motivation is essential to learning and change processes(18) and has been found to influence change

For peer review only

behavior of various physicians.(19-21) In a study on physicians' prescription behavior, Wakefield et al.(22) found that physicians who expressed a commitment to change following participation in a continuing medical education program using interactive small groups were significantly more likely to change their targeted prescription practices in the following half year. Contradictory evidence, however, has also been reported.(23, 24) Palmer(23) assessed the motivation of health professionals to change their practices and found that they improved on tasks for which they reported to have *limited* motivation. This counterintuitive finding can be explained by the significant *system* changes (in addition to individual changes) required to change practices for which professionals' motivation was high. Thus, the relation between motivation and care practice improvement may be less straightforward than originally anticipated.

The reported variability in the effect of motivation on actual care practice change can be explained by attributional processes that impact an individual's motivation to change.(25) For example, during audit and feedback meetings GPs might attribute practice variation to 'the system', particular patients, or fellow healthcare professionals (i.e., hold others responsible for practice variation). In such a case, the GPs are not likely to be inclined to change their own professional practice behavior (cf. (11)). Thus, GPs' attributions are significant in processes of general practice change and need careful consideration in the context of audit and feedback. In our study, therefore, we adopted a attributional perspective on audit and feedback.

In the Dutch context, audit and feedback have become increasingly important for GP professional development. Historically, GPs use pharmacological feedback from pharmacists and their electronic health records to improve prescribing behavior – mainly in educational group sessions. Since the last decade, GPs also use these sessions to discuss diagnostic procedures. At the same time, insurance companies have started to request from GPs information on quality indicators. As GPs expressed a need for audit and feedback sessions based on these quality indicators, the Dutch movement 'Optimale zorg-Dappere dokters' [Optimal care-Daring doctors] initiated an audit and feedback group intervention, aiming to encourage GPs' self-reflection and optimize care.

Group-based critical self-evaluation based on quality indicators is not standard in contemporary quality policies of the medical profession, but can be beneficial to stimulate self-

For peer review only

reflection for optimal care.(12) Understanding how participants respond to the audit and feedback data will facilitate improvement of interventions. To this end, we qualitatively investigated how audit and feedback group sessions contribute to GPs' motivation to change their practice behavior to improve care. We focused on GPs' attributions regarding both content and process of the audit and feedback group sessions by asking:

1. How does the audit and feedback itself contribute to GPs' motivation to change?
2. How does the group discussion contribute to GPs' motivation to change?

For peer review only

THEORETICAL FRAMEWORK

The role of attributions in motivation and behavior change is described in Weiner's Attribution Theory,(26, 27) which indicates that humans "have a tacit goal of understanding and mastering themselves and their environment" and "establish cause-effect relationships for events in their lives".(25) Occurring events lead to attribution, a process of (often subconsciously) seeking an explanation by hypothesizing perceived personal and environmental causes (e.g., ability, effort, luck, task difficulty, mood, health, other people, etc.). These causes can be organized along three causal dimensions: locus (i.e., internal or external to the individual), stability (i.e., stable, fixed or unstable, likely to change), and controllability (i.e., within or outside the individual's control). Based on the specific combination of values in each dimension, occurring events are interpreted as psychologically meaningful responses. As such, the individual's *interpretation* of a particular event determines their response to that event. Typically, bad luck is interpreted as external, unstable and uncontrollable – invoking no or possibly only a passive reaction from the individual; personal effort is internal, changeable, and controllable – and can thus be influenced by an individual's actions; and innate skill is internal, largely fixed, and uncontrollable – making it an unlikely subject of change.(25)

METHODS

Ethics

The Medical Ethics Review Committee of the Amsterdam Academic Medical Centre confirmed that the Medical Research Involving Human Subjects Act does not apply to this study and that an official approval of this study by the committee was not required (reference number W18_200 # 18.241).

Patient and Public Involvement

No patient or public were involved. The development of the research question was informed by two practicing GPs (MH, ISM), who also participated in the design of the study. The results will be disseminated to participants via an email informing GPs about the main results and focusing on issues of future audit and feedback implementation.

Setting

The study was carried out in the region of Amsterdam. Dutch general practice plays an important role in the Dutch health care system, since specialist care in the Netherlands is only accessible upon referral by a GP.(28) All Dutch citizens are registered at a GP-practice in their regional area. Patients visit their GP when faced with a medical problem (except in life-threatening situations). The GP collects and evaluates all relevant medical information. Consequent treatment decisions and referrals to a medical specialist are taken together with the patient. Costs of care are covered by healthcare insurances, which are compulsory for people who live or work in the Netherlands and which include at least basic health care.

Audit and feedback information is gathered and provided to GPs by Vektis, a Dutch center for information and standardization of health insurance. This information is based on declarations of health care costs to insurance companies. It provides data on indicators regarding practice population, consultations, interventions, prescription, and referral rates compared with a standardized Dutch practice, corrected for age, gender, social-economic status and disease severity of the population.

Data collection

We conducted focus group discussions(29, 30) with GPs of four regional GP groups within the Amsterdam region. GP groups interested in this audit and feedback intervention could participate if their practices were in the Amsterdam region. General practitioners were approached and informed about the research purpose and participation practicalities via an information letter. Participating GPs signed informed consent prior to the discussions and sent the audit and feedback information that their practices received from Vektis (data from 2012-2014) to the research team for analysis. The focus group discussions were held between June 2016 and March 2017 at a participating GP's practice, lasted approximately 1.5 hours and were audio-taped for transcription purposes.

For peer review only

Focus group discussions were facilitated by a moderator (ND or MV), who guided the group through the data reports. Resembling Cooke et al.'s intervention(12), an aggregate comparative report of quality indicators (selected for their relevance by each focus group in a session preceding the audit and feedback session) was projected for everyone to see; actual practice information was only available to GPs of that practice, but was shared with other GPs if relevant to the discussion or upon practitioners' own initiation. The facilitator encouraged participants to ask questions or share remarkable or unexpected aspects of their individual feedback information, facilitated interpretation of the feedback data (e.g., by explaining how it is constructed), and probed participants to discuss the consequences of the data for their future daily practice. Each focus group discussion concluded with an exploration of potential issues relevant for follow-up sessions (not reported on in this paper).

The four focus group interactions were transcribed verbatim and anonymized by deleting geographical and personal names. Fragments unintelligible due to simultaneous speech or laughing were transcribed as [unintelligible] and included a timestamp to facilitate fragment location at a later moment of analysis if needed.

Analysis

Transcripts were analyzed using theoretical thematic analysis (31); audiotapes were consulted to improve interpretation where necessary. Key concepts derived from Attribution Theory, e.g. 'external attribution' formed the initial framework for data coding. MB first coded one transcript, supplementing the initial codes with codes derived inductively from the data (e.g., *gaining information* or *evaluation*). MB and ND discussed the coding of this first transcript to ensure coding reliability. Applying constant comparison, several codes were modified or merged for code reduction. Next, MB and ND independently coded a second transcript, adding additional open codes where needed. They discussed their codings in detail until agreement was reached. MB modified the coding of the first two transcripts accordingly and used the final coding scheme to code the two remaining transcripts. Again, MB and ND discussed fragments that could not unambiguously be coded until agreement was reached. Although the content of the last focus groups only partly resembled the content of the first two focus groups, the existing codes sufficed to cover the content. This provided evidence for data saturation. As

For peer review only

a final step, MB organized the code themes into a coherent and internally consistent account of what motivates GPs to change.

Reflexivity

Four members of the research team were medical doctors (MH, ISM, JB, ND), three of whom were practicing GPs (MH, ISM, JB). The practicing GPs approached ND to initiate the study, induced by their practice experience. They did not participate in data collection or analysis to prevent interaction between their individual experience and the data collection process. One of the focus group facilitators was a medical doctor (not practicing). Analysis was primarily done by MB, who has no medical training and therefor was most distant to the content discussed. This benefitted a broad outlook on the data.

RESULTS

Focus groups were attended by 39 GPs from four regional groups (7-10 GPs per focus group). Participants of three focus groups all had practices in an urban area; practices of the fourth focus group's participants were situated in a rural area. GPs' age ranged from 30 to 65 years (the majority being over 50 years of age). Approximately 75 percent of participating GPs work in a practice together with a partner, with the remainder working alone or in a group practice.

Generally, discussions of audit and feedback items commenced with resolving potential interpretation difficulties. Subsequently, GPs construed an understanding of the information, focusing on probable explanations for deviations from average or between practices. These tended to be followed by GPs expressing their motivations to change.

In the following, we first present aspects of the audit and feedback that contributed to GPs' motivation to change behavior. We specifically focus on the attributions that GPs used to explain their hesitation to change. Next, we present aspects of the group discussion that contributed to GPs' motivation to change.

Motivation to change: contribution of audit and feedback information

Two important contributions of audit and feedback emerged from our analysis of the focus group discussions. If anything, the audit and feedback motivated GPs to change *by raising awareness about aspects of their current care practice*. A heightened awareness of one's current practices as

For peer review only

reported in the audit and feedback, could lead to the realization that actual care practices differed extensively from perceived care practices or from the norm. This insight frequently induced GPs to express intentions to further reflect on or take additional steps towards practice change, an example of which is presented in Box 1.

Box 1 *Focus group O*

1	GP A	Well, what I do want to do is check all our cyriax cases to see what the indications were. That is
2		quite a job and it would be very nice if we could receive that audit and feedback. But this
3		finding has already provoked me to look that up in my own Electronic Health Record to see how
4		many have been done.
5	CHA	Yes.
6	GP A	Then we would really have something to talk about, I think.

As this excerpt reveals, being confronted with specific audit and feedback about one's own practice (e.g., the finding that the number of cyriax operations deviates considerably from the norm, line 3) can lead to undertaking specific steps to understand and potentially adapt one's care practices. The audit and feedback further contributes to GPs' motivation to change *by providing indications of the possible impact of change*. The presented audit and feedback displays the degree of deviation from norms. If deviations from 'average' practice are large, changing practice would have a large impact. Minimal deviations from the norm, on the contrary, are judged irrelevant to future practice change. Similarly, the number of patients that are included in a figure signal the impact of potential change. Changes that would affect only a limited number of patients are viewed as trivial, as shown in the excerpt in Box 2.

Box 2 *Focus group P*

1	GP A	There is one other thing that I appreciate about these figures, sometimes deviations are
2		enormous but then it's only about 10 patients, one isn't going to change policy on that.

For these two contributions of audit and feedback to play out, however, GPs pointed out that several conditions have to be met. First, the audit and feedback information *should be reliable and valid*. Suspicion of unreliability of the audit and feedback induces insecurity about possible future actions; one GP said: "it doesn't match with how I feel about it (…), so I don't really know what I should or could do with that". According to another GP, only reliable figures that resemble the GP's own behavior can rightfully trigger change. Besides being reliable, figures should also be valid. If the

For peer review only

reader cannot tell what comprises the figures, it remains unclear why increases and decreases in prescriptions, referrals, and treatments occur and to whom (or what) these changes can be attributed As GP A points out in Box 3, the data's construction is key to its interpretation.

Box 3 *Focus group V*

1	GP A	But then one soon asks oneself: "How did they get to these figures? How is all of this calculated,
2		if the difference is so large, we haven't started working completely differently a year later". So
3		there is something there. That does give a lot of interesting information (…). It always comes
4		down to: What are your norms, why - how are things actually counted? Yes, that's when things
5		get terribly difficult. That says a lot about the reliability.
6	GP B	I would say they have started to count in a different way.

For peer review only

Perceived limited reliability and clarity frequently induced external attributions, i.e. explanations of feedback information by causes external to the GPs influence sphere. If attributed externally, no change talk would follow. As such, unreliability and invalidity of the data compromise the potential contribution of the audit and feedback information to GPs motivation to change care practices.

Second, the audit and feedback information *should be specific.* GPs' motivation to change would benefit from broad themes being split up into smaller subthemes (e.g., ECGs for specific problems instead of one figure for all ECGs made). The contribution of audit and feedback to GPs' motivation to change would be increased if specific prescriptions or patient information would be available on request. This would help understand, for example, extreme prescription rates or costs (as one GP with high costs wondered: "Is that because of that Augmentin for that cat bite?"). Having the option to link prescriptions, referrals, and costs to specific patients would point GPs at potential behavior for change.

Third, audit and feedback information *should be recent and recurrent*. Short term feedback is agreed upon as being critical to the effectiveness of audit and feedback as facilitator of change. GPs do not feel the urge to 'learn' from figures that represent their behavior registered three or more years ago. The feedback should not only be recent, however, but also be recurrent, as Box 4 shows.

Box 4 *Focus group P*

1	GP A	Those figures are very broad and big, really, so I find it difficult to - if one gets back something
2		small from your figures, something that you can improve on easily and if one gets back the
3		figures again after half a year, then I would be more likely to show behavior change.

Fourth, the audit and feedback *should concern GPs' own practices or practices within their own influence sphere*. An example of care practices *outside* the GPs' control are specialist prescriptions. Some GPs suggested to talk to a specialist to discuss deviant figures or refuse particular referrals. More commonly, however, such figures are unlikely subjects for change. GPs would attribute the deviations from 'average' practice represented by these figures to external sources: non-GP health professionals (in case of in-hospital treatment) or other GPs (e.g., if other GPs declare particular treatments erroneously while a participating GP does it correctly, this GP's declaration rates will deviate from the norm despite his declaration practices being 'correct'), individual patients (who might prefer a particular treatment over others), a GP-in-training (whose presence could result in more applications for radiology or lab diagnostics), the practice location (which, for example, might result in fewer home visits if located close to an old-age home or in the care center), regulations (e.g, codes of conduct), or time issues (see Box 5).

Box 5 *Focus group OD*

1	GP A	I do tell them quite often to come - make a new appointment, but I do have too few consults
2		already as well.
3	GP B	Oh so that doesn't help either.
4	GP A	So that doesn't help either.
5	GP C	We cannot even schedule more consults.
6	GP D	Full is full.
7	GP C	Yes, only if you want to continue working through the evening.

Despite being potentially problematic, audit and feedback elements that pertain toissues far beyond GPs' control (i.e., external attribution) do not induce motivation to change their own practices.

Motivation to change: contribution of group discussion

In the current study's audit and feedback sessions, the group contributed to GPs' motivation to change in two ways. Most importantly, the presence of peers *provided a frame of reference* for interpretation and evaluation of feedback figures. During the focus group discussions, GPs could compare their feedback figures. Comparison can be very informative, as one GP points out in Box 6.

Box 6 *Focus group OD*

1	GP A	I think that is interesting, when we see - when I see that I deviate from the national average, and
2		we all deviate, then you think: what do we do about that?

For peer review only

3	GP B	Sure.
4	GP A	If only mine deviates, then I think: well, I have to do something about that.

Apparently, the need (and motivation) to change practices is more pressing if only one GP's practice deviates from the norm compared to deviation common to all participating GPs. Common deviations are often attributed to demographical or geographical characteristics. Comparing each other's audit and feedback thus functions as a filter, isolating idiosyncratic practice-related variation from region-bound practice variation.

Second, the contribution of group members can yield important *insights that participants would not have been able to achieve on their own*. At times, discussing care practices led to an explicitly formulated realization that particular practices had to be adjusted for reasons proposed during the discussion. In the excerpt displayed in Box 7, for instance, one participating GP realized that his hesitation to plan double consults (20 minutes for patients with multiple complaints) instead of the common 10-minute consult (the standard option in Dutch general practice) was unjustified.

Box 7 *Focus group O*

1	GP A	This is such an eye opener for me, you know? I constantly feel guilty when I plan twenty-
2		minute consults twice a day, because then my assistants won't be able to schedule
3		enough patients – that's what I think. But I can only do that!
4	GP B	But darling, we are manning just slightly more patients with twice as many practitioners as you
5		have.
6	GP A	Yes, that's true.
7	GP B	You know, I mean - that is how it is, really.
8	GP C	Yes, so you need an extra doctor, and more double consults.
9	GP A	And a room.
10	GP C	Yes, and if we charge for the double consults as well, then an extra doctor does not cost a thing.
11	GP D	Indeed, then you recover the expenses easily.

For peer review only

Other GPs participating in this interaction are used to planning double consults and do not experience the time issues that GP A mentions in lines 1-2. The reason behind the differences in the number of double consults planned, GP B poses, is probably understaffing (line 4). Following this suggestion, GP C and GP D formulate an explicit need for change (lines 5-6), and potential objections to the proposed change are warded off in lines 7-8. This excerpt shows the benefits of discussing one's audit and feedback with fellow GPs: peers can point out problematic issues or solutions that one has not

considered themselves. Also, peers can encourage each other to explore a solution and motivate each other to change – as we see happens in Box 7.

Notwithstanding their contributions to GPs' motivation to change, however, group discussions sometimes merely initiated sharing of motivations behind and reflections on practice behavior without triggering change talk. This type of motivated sharing of best practices occurred quite frequently throughout the group discussions. In general, though, group discussion is perceived to facilitate interpretation of audit and feedback and evaluations of the need for practice change.

DISCUSSION

The present study qualitatively investigated how audit and feedback group sessions can contribute to GPs' motivation to change practice behavior to improve care. We framed GPs' responses to the group audit and feedback sessions with Attribution Theory. The individual's interpretation of the cause behind a behavior, i.e. whether the cause is internal or external, is stable, and is controllable, is central to this approach. Such interpretation determines the individual's response to that behavior. Understanding GPs' attributions of behavior presented during audit and feedback is therefore essential in the context of designing interventions aimed at changing non-optimal care practices.

The presented analysis of four focus groups with Dutch GPs shows that audit and feedback itself can contribute to motivation to change by raising awareness about current practice. At times, that awareness propels GPs to the next step towards change.(20) Furthermore, audit and feedback can contribute to motivation to change by providing an indication of the potential impact of change in terms of degree of deviation and number of patients, prescriptions, etc. involved. Generally, the lower the impact of change, the lower GPs' motivation to change. Similar to findings reported by Palmer (23), a pragmatic consideration seems to be at play here: although GPs interpret the behavior as controllable (something can be done about it), stable (the behavior does not occur randomly), and located within their own action range (internal locus), the effort does not weigh out the benefit of change. In this respect, GPs' attributions would not explain the contribution of audit and feedback to GPs' motivation to change.

At the same time, however, several conditions under which audit and feedback might be beneficial to motivation to change relate closely to the three attribution dimensions locus, stability, and

For peer review only

controllability. The extent to which the audit and feedback pertain to GPs' *individual, controllable, and changeable* behavior is a strong factor in inducing GPs' expressions of change intention. Behaviors that are attributed to external sources, or fixed or uncontrollable causes (e.g., the system) are an unlikely subject for behavior change.(25) Additionally, audit and feedback ought to be *reliable*, *valid, specific*, *recent* and *recurrent* to potentially effectuate change. This finding endorses the cardinal importance of high quality audit and feedback discussed in non-GP audit and feedback settings (7-9) and confirms a number of recommendations for effective audit and feedback presented by Brehaut and colleagues(11). Simultaneously, and more practically, these results indicate that significant adjustment of contemporary Dutch audit and feedback is required to assure prompt and profitable use for GP professionalization.

Finally, our findings point to the key role of collectively discussing audit and feedback. As indicated by Trietsch et al., social influence and norms affect participants' reflective behavior and corresponding intentions to change current practice during peer interaction.(32) Contrasting with Ivers et al.'s conclusion that there is very limited evidence for peer-comparison audit and feedback being either more or less effective than individual performance information,(6) our data show that peer comparison in general and group processes in particular stimulate critical appraisal of the audit and feedback and the need for behavioral change.(12, 33-35) Peer comparison provides an interpretative framework for individual practice data and peer interactivity provides ample opportunities to explore alternative practices and promising avenues for improvement.(5, 10)

Practically, our findings can inform audit and feedback practice in the Dutch context by identifying ways to improve the reports and the way they are delivered. Optionally, already accessible practice-related information (e.g., in the Electronic Health Record) can be employed as a starting point for an informative, easily accessible, and adaptively employable application serving GP professionalization. Additionally, frequent meetings with GPs practicing in the same local or regional area to facilitate group reflection and discussion would be very valuable.

The present investigation of GPs' motivations to change based on audit and feedback group sessions offers a detailed understanding of GPs' change considerations. Member checking confirmed the recognizability and usefulness of the findings. By conducting focus group discussions based on

For peer review only

personal and comparative audit and feedback, we were able to tap into real-time communicated reflective processes and sketch a comprehensive image of the diversity of attributions and factors impacting GPs' motivation to change. Yet, the image could be blurred in two ways. The peer group setting, despite being beneficial to change motivation, might have induced participants to want to look their best. Besides, expressed motivation to change is no guarantee for actual change.(22, 36). Therefore, future work exploring the effects of audit and feedback on diverse aspects of care quality (patient health, patient satisfaction, costs) – both in the short and the long term – is crucial to determining the effectiveness of this audit and feedback tool for GP professionalization. The use of a specific type of audit and feedback demands cautious interpretations as far as results relating to features of this specific type of audit and feedback are concerned. Yet, the current study's attributional perspective on audit and feedback has certainly enriched our understanding of the complexities of those processes that jointly foster GPs' professionalization: individual reflection and critical discussion.

For peer review only

Contributors

All authors contributed substantially to the conception, design, or execution of the reported study. MH and ISM, in collaboration with ND, took initiative for the study. ND, MV, MH and ISM designed the study and collected the data. JB participated in the interpretation of the data; MVB, ND and MV participated in data analysis. MVB was responsible for the write-up of the study. All authors critically revised its content and provided final approval of the version to be published.

Funding

This research received no specific grant from any funding agency in the public, commercial or not-for-profit sectors.

Competing interests

None declared.

Data sharing

The data for this paper may be obtained from the authors upon request.

For peer review only

References

1. Koefoed MM, Søndergaard J, Christensen Rd, et al. General practice variation in spirometry testing among patients receiving first-time prescriptions for medication targeting obstructive lung disease in Denmark: a population-based observational study. *BMC Fam Pract* 2013;14:113.

2. Millett C, Car J, Eldred D, et al. Diabetes prevalence, process of care and outcomes in relation to practice size, caseload and deprivation: national cross-sectional study in primary care. *J R Soc Med* 2007;100(6):275-83.

3. Nielen MMJ, Schellevis FG, Verheij RA. Inter-practice variation in diagnosing hypertension and diabetes mellitus: a cross-sectional study in general practice. *BMC Fam Pract* 2009;10:6.

4. Verstappen WM, van der Weijden T, Sijbrandij J, et al. Effect of a practice-based strategy on test ordering performance of primary care physicians: A randomized trial. *JAMA* 2003;289(18):2407-12.

5. Cervero RM, Gaines JK. The Impact of CME on Physician Performance and Patient Health Outcomes: An Updated Synthesis of Systematic Reviews. *J Contin Educ Health Prof* 2015;35(2):131-8.

6. Ivers N, Jamtvedt G, Flottorp S, et al. Audit and feedback: effects on professional practice and health care outcomes. *Cochrane Database Syst Rev* 2012(6):1-227.

7. O'Connell DL, Henry D, Tomlins R. Randomised Controlled Trial of Effect of Feedback on General Practitioners' Prescribing in Australia. *BMJ* 1999;318(7182):507-11.

8. Mugford M, Banfield P, O'Hanlon M. Effects Of Feedback Of Information On Clinical Practice: A Review. *BMJ* 1991;303(6799):398-402.

9. Axt-Adam P, van der Wouden JC, van der Does E. Influencing Behavior of Physicians Ordering Laboratory Tests: A Literature Study. *Med Care* 1993;31(9):784-94.

10. Verstappen WHJM, van der Weijden T, Dubois WI, et al. Improving Test Ordering in Primary Care: The Added Value of a Small-Group Quality Improvement Strategy Compared With Classic Feedback Only. *Ann Fam Med* 2004;2(6):569-75.

11. Brehaut JC, Colquhoun HL, Eva KW, et al. Practice feedback interventions: 15 suggestions for optimizing effectiveness. *Ann Intern Med* 2016;164(6):435-41.

For peer review only

12. Cooke LJ, Duncan D, Rivera L, et al. How do physicians behave when they participate in audit and feedback activities in a group with their peers? *Implement Sci* 2018;13(1):104.

13. Kluger AN, DeNisi A. The effects of feedback interventions on performance: A historical review, a meta-analysis, and a preliminary feedback intervention theory. *Psychol Bull* 1996;119(2):254.

14. Steele CM. The psychology of self-affirmation: Sustaining the integrity of the self. *Adv Exp Soc Psych* 1988:21:261-302.

15. Damschroder LJ, Aron DC, Keith RE, et al. Fostering implementation of health services research findings into practice: a consolidated framework for advancing implementation science. *Implement Sci* 2009;4(1):50.

16. Bandura A. Social learning theory. New Jersey: Prentice Hall; 1977.

17. Brown B. Harnessing opportunities for quality improivement from primary care electronic health records [PhD dissertation]: University of Manchester; 2018.

18. Kusurkar RA, Artino AR, Ten Cate TJ. Motivation and learning. In: Bhuiyan PS, Rege NN, Supe A, editors. The art of teaching medical students. New Delhi: Reed Elsevier India Pvt. Ltd. 2015:62-76.

19. Fox R, Mazmanian P, Putnam RW. Changing and learning in the lives of physicians. New York: Praeger; 1989.

20. Grol R, Wensing M. What drives change? Barriers to and incentives for achieving evidence-based practice. *Med J Aus* 2004;180(6 Suppl):S57-60.

21. Kanouse DE, Jacoby I. When does information change practitioners' behavior? *Int J Technol Assess Health Care* 1988;4(1):27-33.

22. Wakefield J, Herbert CP, Maclure M, et al. Commitment to change statements can predict actual change in practice. *J Contin Educ Health Prof* 2003;23(2):81-92.

23. Palmer RH, Louis TA, Hsu L-N, et al. A Randomized Controlled Trial of Quality Assurance in Sixteen Ambulatory Care Practices. *Med Care* 1985;23(6):751-70.

24. Foy R, MacLennan G, Grimshaw J, et al. Attributes of clinical recommendations that influence change in practice following audit and feedback. *J Clin Epidemiol* 2002;55(7):717-22.

25. Cook DA, Artino AR. Motivation to learn: an overview of contemporary theories. *Med Educ* 2016;50(10):997-1014.

For peer review only

26. Weiner B. An Attributional Theory of Achievement Motivation and Emotion. *Psychol Rev* 1985;92(4):548-73.

27. Weiner B. Intrapersonal and Interpersonal Theories of Motivation from an Attributional Perspective. *Educ Psychol Rev* 2000;12(1):1-14.

28. Vektis. Zorgprisma Publiek. Huisartsenzorg. 2017. https://www.zorgprismapubliek.nl/informatie-over/huisartsenzorg/huisartsenzorg/ (accessed June 2017).

29. Krueger RA, Casey MA. Focus groups. A practical guide for applied research. Thousand Oaks, CA: SAGE Publications, Inc. 2000.

30. Tong A, Sainsbury P, Craig J. Consolidated criteria for reporting qualitative research (COREQ): a 32-item checklist for interviews and focus groups. *Int J Qual Health Care* 2007;19(6):349-57.

31. Braun V, Clarke V. Using thematic analysis in psychology. *Qual Res Psychol* 2006;3(2):77-101.

32. Trietsch J, van der Weijden T, Verstappen W, et al. A cluster randomized controlled trial aimed at implementation of local quality improvement collaboratives to improve prescribing and test ordering performance of general practitioners: Study Protocol. *Implement Sci* 2009;4:6.

33. Sargeant J, Lockyer J, Mann K, et al. Facilitated reflective performance feedback: developing an evidence-and theory-based model that builds relationship, explores reactions and content, and coaches for performance change (R2C2). *Acad Med* 2015;90(12):1698-706.

34. Carless D, Salter D, Yang M, et al. Developing sustainable feedback practices. *Stud High Educ* 2011;36(4):395-407.

35. Cooke LJ, Duncan D, Rivera L, et al. The Calgary Audit and Feedback Framework: a practical, evidence-informed approach for the design and implementation of socially constructed learning interventions using audit and group feedback. *Implement Sci* 2018;13(1):136.

36. Overton GK, McCalister P, Kelly D, et al. Practice-based Small Group Learning: How health professionals view their intention to change and the process of implementing change in practice. *Med Teach* 2009;31(11):e514-e20.

For peer review only

SRQR checklist (O'Brien et al., 2014)

Item	Place in manuscript (clean version)
Title	p1, l1-2
Abstract	p2
Problem formulation	p4, par1+2
Purpose or research question	p6, l2-7
Qualitative approach and research paradigm	p8, l1 under 'Analysis'
Researcher characteristics and reflexivity	p9, under 'Reflexivity'
Context	p7, under 'Setting'
Sampling strategy	p7, l2-4 under 'Data collection'
Ethical issues pertaining to human subjects	p6, under 'Ethics'
Data collection methods	p7-8, under 'Data collection'
Data collection instruments and technologies	p7-8, under 'Data collection'
Units of study	p9, l1-5 under 'Results'
Data processing	p8, l11-14
Data analysis	p8, under 'Analysis'
Techniques to enhance trustworthiness	p15, par4, l2-3
Synthesis and interpretation	p9-14, under 'Results'
Links to empirical data	p10-13, Boxes 1-7
Integration with prior work, implications, transferability, and contribution(s) to the field	p14-16, under 'Discussion'
Limitations	p16, l3-5 (and also p3)
Conflicts of interest	p17, under 'Competing interests'
Funding	p17, under 'Funding'

For peer review only

BMJ Open

What motivates General Practitioners to change practice behavior? A qualitative study of audit and feedback group sessions in Dutch General Practice

Journal:	*BMJ Open*
Manuscript ID	bmjopen-2018-025286.R2
Article Type:	Research
Date Submitted by the Author:	01-Mar-2019
Complete List of Authors:	van Braak, Marije; Erasmus Medical Centre, General Practice<br>Visser, Mechteld; Amsterdam University Medical Centre, General Practice<br>Holtrop, Marije; General Practitioners Holtrop and Sieben<br>Statius Muller, Ilona; Ubbens and Statius Muller General Practitioners<br>Bont, Jettie; Amsterdam University Medical Centre, General Practice<br>van Dijk, Nynke; Amsterdam University Medical Centre, General Practice
**Primary Subject Heading**:	General practice / Family practice
Secondary Subject Heading:	Medical education and training, Qualitative research
Keywords:	MEDICAL EDUCATION & TRAINING, PRIMARY CARE, QUALITATIVE RESEARCH

SCHOLARONE™
Manuscripts

What motivates General Practitioners to change practice behavior? A qualitative study of audit and feedback group sessions in Dutch General Practice

Correspondence:

Marije van Braak, MSc

Department of General Practice, Erasmus Medical Centre

Postbus 2040, Dr. Molewaterplein 40, 3015 GD Rotterdam, the Netherlands

m.vanbraak@erasmusmc.nl

+31 10 7042518

Mechteld R. M. Visser, PhD

Department of General Practice, Amsterdam University Medical Centre, Amsterdam, the Netherlands

Marije Holtrop, MD, GP

General Practitioners Holtrop and Sieben

Amsterdam, the Netherlands

Ilona Statius Muller, MD, GP

Ubbens and Statius Muller General Practitioners

Amsterdam, the Netherlands

Jettie Bont, MD, PhD, GP

Department of General Practice, Amsterdam University Medical Centre, Amsterdam, the Netherlands

Nynke van Dijk, Professor, MD, PhD

Department of General Practice, Amsterdam University Medical Centre, Amsterdam, the Netherlands

Word count: 4064

For peer review only

ABSTRACT

Objectives Adopting an attributional perspective, the current article investigates how audit and feedback group sessions contribute to GPs' motivation to change their practice behavior to improve care. We focus on the contributions of the audit and feedback itself (content) and the group discussion (process).

Methods Four focus groups, comprising a total of 39 participating Dutch GPs, discussed and compared audit and feedback of their practices. The focus groups were analyzed thematically.

Results Audit and feedback contributed to GPs' motivation to change in two ways: by raising awareness about aspects of their current care practice and by providing indications of the possible impact of change. For these contributions to play out, the audit and feedback should be reliable and valid, specific, recent and recurrent, and concern GPs' own practices or practices within their own influence sphere. Care behavior attributed to external, uncontrollable, or unstable causes would not induce change. The added value of the group is twofold as well: group discussion contributed to GPs' motivation to change by providing a frame of reference and by affording insights that participants would not have been able to achieve on their own.

Conclusions In audit and feedback group sessions, both audit and feedback information and group discussion can valuably contribute to GPs' motivation to change care practice behavior. Peer interaction can positively contribute to explore alternative practices and avenues for improvement. Local or regional peer meetings would be beneficial in facilitating reflection and discussion. An important avenue for future studies is to explore the contribution of audit and feedback and small-group discussion to *actual* practice change.

For peer review only

For peer review only - http://bmjopen.bmj.com/site/about/guidelines.xhtml

STRENGTHS AND LIMITATIONS OF THIS STUDY

- Framed within Attribution Theory, the study provides a novel perspective on audit and feedback.

- Focus group discussions on personal and comparative audit and feedback allowed us to tap into real-time reflective processes.

- Qualitative analysis of recorded interaction between GPs allowed for detailed insight into the value of peer interaction in discussions on practice change.

- Voluntary GP participation may have resulted in a sample of participants with a special interest in audit and feedback and behavior change.

- The study focused only on effects of audit and feedback and group discussion on intended, not actual, practice change.

For peer review only

INTRODUCTION

In taking the Hippocratic oath, general practitioners – and other doctors as well – express their intention to treat patients to the best of their ability. Yet, care practices of general practitioners show substantial unintended variation.(1-3) Part of this diversity is induced by external practice or population factors, such as practice size.(2) Individual factors also play a role: general practitioners' knowledge, skills, experience, interests and preferences can induce between-practice variation. This variation might be related to differences in clinical judgment based on considerations of evidence, clinical experience and patient preferences,(4) but sometimes is not intended and may lead to lower patient care quality.

For general practitioners (GPs) to develop their professional practice, they should be aware of this unintended variation. Reflection on between-practice variation can lead to adjustments in professional care behavior, eventually improving the quality of patient care.(5) Audit and feedback on GPs' performance can effectively improve professional practice, albeit under certain optimally-designed conditions and in the right context.(6) Several factors influencing the effectiveness of feedback have been researched. These include the level of feedback detail,(7) its timing, (8, 9) and the interactivity of the feedback-giving process.(4, 5, 9, 10) Based on expert interviews, systematic reviews, and experience, Brehaut and colleagues suggest that practice feedback interventions can be optimized by, amongst others, linking it to established goals, providing feedback in more than one way, minimizing extraneous cognitive load for feedback recipients, increasing the credibility of the data and preventing defensive reactions to feedback.(11) Additionally, recent research on group audit and feedback points at the added value of socially constructed learning activities in audit and feedback group sessions.(12)

The theoretical framing of audit and feedback research is diverse, ranging from feedback theories (e.g., Feedback Intervention Theory, (13)) to psychological theories (e.g. Self Affirmation Theory, (14)) to implementation theories (e.g. Consolidated framework for implementation research, (15)) to learning theories (e.g. Social Learning Theory, (16)).(17) In our study, we explicitly focus on GPs' *motivation* to change. This perspective on audit and feedback has not been used before. Yet, motivation is essential to learning and change processes(18) and has been found to influence change

For peer review only

behavior of various physicians.(19-21) In a study on physicians' prescription behavior, Wakefield et al.(22) found that physicians who expressed a commitment to change following participation in a continuing medical education program using interactive small groups were significantly more likely to change their targeted prescription practices in the following half year. Contradictory evidence, however, has also been reported.(23, 24) Palmer(23) assessed the motivation of health professionals to change their practices and found that they improved on tasks for which they reported to have *limited* motivation. This counterintuitive finding can be explained by the significant *system* changes (in addition to individual changes) required to change practices for which professionals' motivation was high. Thus, the relation between motivation and care practice improvement may be less straightforward than originally anticipated.

The reported variability in the effect of motivation on actual care practice change can be explained by attributional processes that impact an individual's motivation to change.(25) For example, during audit and feedback meetings GPs might attribute practice variation to 'the system', particular patients, or fellow healthcare professionals (i.e., hold others responsible for practice variation). In such a case, the GPs are not likely to be inclined to change their own professional practice behavior (cf. (11)). Thus, GPs' attributions are significant in processes of general practice change and need careful consideration in the context of audit and feedback. In our study, therefore, we adopted a attributional perspective on audit and feedback.

In the Dutch context, audit and feedback have become increasingly important for GP professional development. Historically, GPs use pharmacological feedback from pharmacists and their electronic health records to improve prescribing behavior – mainly in educational group sessions. Since the last decade, GPs also use these sessions to discuss diagnostic procedures. At the same time, insurance companies have started to request from GPs information on quality indicators. As GPs expressed a need for audit and feedback sessions based on these quality indicators, the Dutch movement 'Optimale zorg-Dappere dokters' [Optimal care-Daring doctors] initiated an audit and feedback group intervention, aiming to encourage GPs' self-reflection and optimize care.

Group-based critical self-evaluation based on quality indicators is not standard in contemporary quality policies of the medical profession, but can be beneficial to stimulate self-

For peer review only

reflection for optimal care.(12) Understanding how participants respond to the audit and feedback data will facilitate improvement of interventions. To this end, we qualitatively investigated how audit and feedback group sessions contribute to GPs' motivation to change their practice behavior to improve care. We focused on GPs' attributions regarding both content and process of the audit and feedback group sessions by asking:

1. How does the audit and feedback itself contribute to GPs' motivation to change?
2. How does the group discussion contribute to GPs' motivation to change?

THEORETICAL FRAMEWORK

The role of attributions in motivation and behavior change is described in Weiner's Attribution Theory,(26, 27) which indicates that humans "have a tacit goal of understanding and mastering themselves and their environment" and "establish cause-effect relationships for events in their lives".(25) Occurring events lead to attribution, a process of (often subconsciously) seeking an explanation by hypothesizing perceived personal and environmental causes (e.g., ability, effort, luck, task difficulty, mood, health, other people, etc.). These causes can be organized along three causal dimensions: locus (i.e., internal or external to the individual), stability (i.e., stable, fixed or unstable, likely to change), and controllability (i.e., within or outside the individual's control). Based on the specific combination of values in each dimension, occurring events are interpreted as psychologically meaningful responses. As such, the individual's *interpretation* of a particular event determines their response to that event. Typically, bad luck is interpreted as external, unstable and uncontrollable – invoking no or possibly only a passive reaction from the individual; personal effort is internal, changeable, and controllable – and can thus be influenced by an individual's actions; and innate skill is internal, largely fixed, and uncontrollable – making it an unlikely subject of change.(25)

METHODS

Ethics

The Medical Ethics Review Committee of the Amsterdam Academic Medical Centre confirmed that the Medical Research Involving Human Subjects Act does not apply to this study and that an official approval of this study by the committee was not required (reference number W18_200 # 18.241).

Patient and Public Involvement

For peer review only

No patient or public were involved. The development of the research question was informed by two practicing GPs (MH, ISM), who also participated in the design of the study. The results will be disseminated to participants via an email informing GPs about the main results and focusing on issues of future audit and feedback implementation.

Setting

The study was carried out in the region of Amsterdam. Dutch general practice plays an important role in the Dutch health care system, since specialist care in the Netherlands is only accessible upon referral by a GP.(28) All Dutch citizens are registered at a GP-practice in their regional area. Patients visit their GP when faced with a medical problem (except in life-threatening situations). The GP collects and evaluates all relevant medical information. Consequent treatment decisions and referrals to a medical specialist are taken together with the patient. Costs of care are covered by healthcare insurances, which are compulsory for people who live or work in the Netherlands and which include at least basic health care.

Audit and feedback information is gathered and provided to GPs by Vektis, a Dutch center for information and standardization of health insurance. This information is based on declarations of health care costs to insurance companies. It provides data on indicators regarding practice population, consultations, interventions, prescription, and referral rates compared with a standardized Dutch practice, corrected for age, gender, social-economic status and disease severity of the population.

Data collection

We conducted focus group discussions(29, 30) with GPs of four regional GP groups within the Amsterdam region. GP groups interested in this audit and feedback intervention could participate if their practices were in the Amsterdam region. General practitioners were approached and informed about the research purpose and participation practicalities via an information letter. Participating GPs signed informed consent prior to the discussions and sent the audit and feedback information that their practices received from Vektis (data from 2012-2014) to the research team for analysis. The focus group discussions were held between June 2016 and March 2017 at a participating GP's practice, lasted approximately 1.5 hours and were audio-taped for transcription purposes.

For peer review only

Focus group discussions were facilitated by a moderator (ND or MV), who guided the group through the data reports. Resembling Cooke et al.'s intervention(12), an aggregate comparative report of quality indicators (selected for their relevance by each focus group in a session preceding the audit and feedback session) was projected for everyone to see; actual practice information was only available to GPs of that practice, but was shared with other GPs if relevant to the discussion or upon practitioners' own initiation. The facilitator encouraged participants to ask questions or share remarkable or unexpected aspects of their individual feedback information, facilitated interpretation of the feedback data (e.g., by explaining how it is constructed), and probed participants to discuss the consequences of the data for their future daily practice. Each focus group discussion concluded with an exploration of potential issues relevant for follow-up sessions (not reported on in this paper).

The four focus group interactions were transcribed verbatim and anonymized by deleting geographical and personal names. Fragments unintelligible due to simultaneous speech or laughing were transcribed as [unintelligible] and included a timestamp to facilitate fragment location at a later moment of analysis if needed.

Analysis

Transcripts were analyzed using theoretical thematic analysis (31); audiotapes were consulted to improve interpretation where necessary. Key concepts derived from Attribution Theory, e.g. 'external attribution' formed the initial framework for data coding. MB first coded one transcript, supplementing the initial codes with codes derived inductively from the data (e.g., *gaining information* or *evaluation*). MB and ND discussed the coding of this first transcript to ensure coding reliability. Applying constant comparison, several codes were modified or merged for code reduction. Next, MB and ND independently coded a second transcript, adding additional open codes where needed. They discussed their codings in detail until agreement was reached. MB modified the coding of the first two transcripts accordingly and used the final coding scheme to code the two remaining transcripts. Again, MB and ND discussed fragments that could not unambiguously be coded until agreement was reached. Although the content of the last focus groups only partly resembled the content of the first two focus groups, the existing codes sufficed to cover the content. This provided evidence for data saturation. As

For peer review only

a final step, MB organized the code themes into a coherent and internally consistent account of what motivates GPs to change.

Reflexivity

Four members of the research team were medical doctors (MH, ISM, JB, ND), three of whom were practicing GPs (MH, ISM, JB). The practicing GPs approached ND to initiate the study, induced by their practice experience. They did not participate in data collection or analysis to prevent interaction between their individual experience and the data collection process. One of the focus group facilitators was a medical doctor (not practicing). Analysis was primarily done by MB, who has no medical training and therefor was most distant to the content discussed. This benefitted a broad outlook on the data.

For peer review only

RESULTS

Focus groups were attended by 39 GPs from four regional groups (7-10 GPs per focus group). Participants of three focus groups all had practices in an urban area; practices of the fourth focus group's participants were situated in a rural area. GPs' age ranged from 30 to 65 years (the majority being over 50 years of age). Approximately 75 percent of participating GPs work in a practice together with a partner, with the remainder working alone or in a group practice.

Generally, discussions of audit and feedback items commenced with resolving potential interpretation difficulties. Subsequently, GPs construed an understanding of the information, focusing on probable explanations for deviations from average or between practices. These tended to be followed by GPs expressing their motivations to change.

In the following, we first present aspects of the audit and feedback that contributed to GPs' motivation to change behavior. We specifically focus on the attributions that GPs used to explain their hesitation to change. Next, we present aspects of the group discussion that contributed to GPs' motivation to change.

Motivation to change: contribution of audit and feedback information

Two important contributions of audit and feedback information to GPs' motivation to change emerged from our analysis of the focus group discussions. First, the audit and feedback motivated GPs to change *by raising awareness about aspects of their current care practice*. A heightened awareness

of one's current practices as reported in the audit and feedback, could lead to the realization that actual care practices differed extensively from perceived care practices or from the norm. This insight frequently induced GPs to express intentions to further reflect on or take additional steps towards practice change, an example of which is presented in Box 1.

Box 1 *Focus group O*

1	GP A	Well, what I do want to do is check all our cyriax cases [i.e., orthopaedic corticosteroid
2		injections] to see what the indications were. That is quite a job and it would be very nice if we
3		could receive that audit and feedback. But this finding has already provoked me to look that up
4		in my own Electronic Health Record to see how many have been done.
5	CHA	Yes.
6	GP A	Then we would really have something to talk about, I think.

For peer review only

As this excerpt reveals, being confronted with specific audit and feedback about one's own practice (e.g., the finding that the number of cyriax cases, for example a corticosteroid knee injection, deviates considerably from the norm, line 3) can lead to undertaking specific steps to understand and potentially adapt one's care practices.

Second, the audit and feedback further contributes to GPs' motivation to change *by providing insight into the degree of deviation from norms*. If deviations from 'average' practice are large, changing practice would have a large impact. Minimal deviations from the norm, on the contrary, are judged irrelevant to future practice change. Similarly, the number of patients that are included in a figure signal the impact of potential change. Deviations – either negative or positive – in practice behavior were less likely a driver for change if only a few patients were involved (Box 2).

Box 2 *Focus group P*

1	GP A	There is one other thing that I appreciate about these figures, sometimes deviations are
2		enormous but then it's only about 10 patients, one isn't going to change policy on that.

For these two contributions of audit and feedback to play out, however, GPs pointed out that several conditions have to be met. First, the audit and feedback information *should be reliable and valid*. Suspicion of unreliability of the audit and feedback induces insecurity about possible future actions; one GP said: "it doesn't match with how I feel about it (…), so I don't really know what I should or could do with that". According to another GP, only reliable figures that resemble the GP's own behavior can rightfully trigger change. Besides being reliable, figures should also be valid.

Examples of information that GPs considered to be invalid are: prescriptions that were recorded as prescribed by the GP, but were in fact specialist prescriptions, figures that simply *could not be true* (e.g. only three recorded prescriptions of a medicine that is very commonly prescribed), or drastic changes in particular prescription behavior from one year to another, while prescription policies were unchanged. If the reader cannot tell what comprises the figures, it remains unclear why increases and decreases in prescriptions, referrals, and treatments occur and to whom (or what) these changes can be attributed. As GP A points out in Box 3, the data's construction is key to its interpretation.

Box 3 *Focus group V*

1	GP A	But then one soon asks oneself: "How did they get to these figures? How is all of this calculated,
2		if the difference is so large, we haven't started working completely differently a year later". So
3		there is something there. That does give a lot of interesting information (…). It always comes
4		down to: What are your norms, why - how are things actually counted? Yes, that's when things
5		get terribly difficult. That says a lot about the reliability.
6	GP B	I would say they have started to count in a different way.

Perceived limited reliability and clarity frequently induced external attributions, i.e. explanations of feedback information by causes external to the GPs influence sphere. If attributed externally, no change talk would follow. As such, unreliability and invalidity of the data compromise the potential contribution of the audit and feedback information to GPs motivation to change care practices.

Second, the audit and feedback information *should be specific.* GPs' motivation to change would benefit from broad themes being split up into smaller subthemes (e.g., ECGs for specific problems instead of one figure for all ECGs made). The contribution of audit and feedback to GPs' motivation to change would be increased if specific prescriptions or patient information would be available on request. This would help understand, for example, extreme prescription rates or costs (as one GP with high costs wondered: "Is that because of that Augmentin for that cat bite?"). Having the option to link prescriptions, referrals, and costs to specific patients would point GPs at potential behavior for change.

Third, audit and feedback information *should be recent and recurrent*. Short term feedback is agreed upon as being critical to the effectiveness of audit and feedback as facilitator of change. GPs do not feel the urge to 'learn' from figures that represent their behavior registered three or more years ago. The feedback should not only be recent, however, but also be recurrent, as Box 4 shows.

For peer review only

Box 4 *Focus group P*
1 GP A Those figures are very broad and big, really, so I find it difficult to - if one gets back something
2 small from your figures, something that you can improve on easily and if one gets back the
3 figures again after half a year, then I would be more likely to show behavior change.

Fourth, the audit and feedback *should concern GPs' own practices or practices within their own influence sphere*. An example of care practices *outside* the GPs' control are specialist prescriptions. Some GPs suggested to talk to a specialist to discuss deviant figures or refuse particular referrals. More commonly, however, such figures are unlikely subjects for change. GPs would attribute the deviations from 'average' practice represented by these figures to external sources. Examples of such sources are non-GP health professionals (in case of in-hospital treatment) a GP-in-training (whose presence could result in more applications for radiology or lab diagnostics), the practice location (which, for example, might result in fewer home visits if located close to an old-age home or in the care center), regulations (e.g, codes of conduct), and time issues (see Box 5).

Box 5 *Focus group OD*
1 GP A I do tell them quite often to come - make a new appointment, but I do have too few consults
2 already as well.
3 GP B Oh so that doesn't help either.
4 GP A So that doesn't help either.
5 GP C We cannot even schedule more consults.
6 GP D Full is full.
7 GP C Yes, only if you want to continue working through the evening.

Despite being potentially problematic, audit and feedback elements that pertain to issues far beyond GPs' control (i.e., external attribution) do not induce motivation to change their own practices.

Motivation to change: contribution of group discussion

In the current study's audit and feedback sessions, the group contributed to GPs' motivation to change in two ways. First, and most importantly, the presence of peers *provided a frame of reference* for interpretation and evaluation of feedback figures. During the focus group discussions, GPs could compare their feedback figures. Comparison can be very informative, as one GP points out in Box 6.

Box 6 *Focus group OD*
1 GP A I think that is interesting, when we see - when I see that I deviate from the national average, and
2 we all deviate, then you think: what do we do about that?
3 GP B Sure.

For peer review only

4	GP A	If only mine deviates, then I think: well, I have to do something about that.

Apparently, the need (and motivation) to change practices is more pressing if only one GP's practice deviates from the norm compared to deviation common to all participating GPs. Common deviations are often attributed to demographical or geographical characteristics. Comparing each other's audit and feedback thus functions as a filter, isolating idiosyncratic practice-related variation from region-bound practice variation.

Second, the contribution of group members can yield important *insights that participants would not have been able to achieve on their own*. At times, discussing care practices led to an explicitly formulated realization that particular practices had to be adjusted for reasons proposed during the discussion. In the excerpt displayed in Box 7, for instance, one participating GP realized that his hesitation to plan double consults (20 minutes for patients with multiple complaints) instead of the common 10-minute consult (the standard option in Dutch general practice) was unjustified.

Box 7 *Focus group O*

1	GP A	This is such an eye opener for me, you know? I constantly feel guilty when I plan twenty-
2		minute consults twice a day, because then my assistants won't be able to schedule
3		enough patients – that's what I think. But I can only do that!
4	GP B	But darling, we are manning just slightly more patients with twice as many practitioners as you
5		have.
6	GP A	Yes, that's true.
7	GP B	You know, I mean - that is how it is, really.
8	GP C	Yes, so you need an extra doctor, and more double consults.
9	GP A	And a room.
10	GP C	Yes, and if we charge for the double consults as well, then an extra doctor does not cost a thing.
11	GP D	Indeed, then you recover the expenses easily.

Other GPs participating in this interaction are used to planning double consults and do not experience the time issues that GP A mentions in lines 1-2. The reason behind the differences in the number of double consults planned, GP B poses, is probably understaffing (line 4). Following this suggestion, GP C and GP D formulate an explicit need for change (lines 5-6), and potential objections to the proposed change are warded off in lines 7-8. This excerpt shows the benefits of discussing one's audit and feedback with fellow GPs: peers can point out problematic issues or solutions that one has not considered themselves. Also, peers can encourage each other to explore a solution and motivate each other to change – as we see happens in Box 7.

For peer review only

Notwithstanding their contributions to GPs' motivation to change, however, group discussions sometimes merely initiated sharing of motivations behind and reflections on practice behavior without triggering change talk. This type of motivated sharing of best practices occurred quite frequently throughout the group discussions. In general, though, group discussion is perceived to facilitate interpretation of audit and feedback and evaluations of the need for practice change.

DISCUSSION

The present study qualitatively investigated how audit and feedback group sessions can contribute to GPs' motivation to change practice behavior to improve care. We framed GPs' responses to the group audit and feedback sessions with Attribution Theory. This theory contends that an individual's motivation to change behavior is contingent on their interpretation of the cause behind that behavior, i.e. whether the cause is internal or external, is stable, and is controllable, is central to this approach. Understanding GPs' attributions of behavior presented during audit and feedback is therefore essential for designing interventions aimed at changing suboptimal care practices.

The presented analysis shows that audit and feedback information can contribute to motivation to change by raising awareness about current practice. At times, that awareness propels GPs to the next step towards change.(20) Audit and feedback can also contribute to motivation to change by providing an indication of the potential impact of change in terms of degree of deviation and number of patients, prescriptions, etc. involved. Generally, the lower the impact of change, the lower GPs' motivation to change. A pragmatic consideration seems to be at play here.(23) Even if GPs interpret the behavior as controllable (something can be done about it), stable (it does not occur randomly), and within their own action range (internal locus), the effort does not outweigh the benefit of change. In these cases, GPs' attributions would not explain the contribution of audit and feedback to GPs' motivation to change. In general, though, the extent to which the audit and feedback pertain to GPs' *individual, controllable, and changeable* behavior is a strong factor in inducing GPs' expressions of change intention – in line with the tenet of Attribution Theory.(25)

Our findings also point to the key role of *collectively discussing* audit and feedback. As indicated by Trietsch et al., social influence and norms affect participants' reflective behavior and corresponding intentions to change current practice during peer interaction.(32) Whereas Ivers et al.

For peer review only

conclude that there is very limited evidence for peer-comparison audit and feedback being either more or less effective than individual performance information,(6) our data show that peer comparison in general and group processes in particular stimulate critical appraisal of the audit and feedback and the need for behavioral change.(12, 33-35) Peer comparison provides an interpretative framework for individual practice data and peer interactivity provides ample opportunities to explore alternative practices and promising avenues for improvement.(5, 10)

Practically, our findings indicate that significant adjustment of contemporary Dutch audit and feedback practices is required to assure prompt and profitable use for improvement of professional practice. To potentially effectuate change, audit and feedback ought to be *reliable*, *valid, specific*, *recent* and *recurrent* (cf. recommendations in similar and other research contexts(7-9)(11)). We suggest to employ already accessible practice-related information (e.g., in the Electronic Health Record) as a starting point for an informative, easily accessible, and adaptively employable application serving improvement of GPs' professional practice. Additionally, frequent meetings with GPs practicing in the same local or regional area beyond the one currently investigated would be very valuable to facilitate group reflection and discussion across the country. This would promote self-governance of the Dutch GPs, in appreciation of the common needs that led to the Optimal Care-Daring Doctors movement that started this audit and feedback initiative. Future evaluation research on such interventions would be essential to ensure progressive refinement of the intervention.

By conducting focus group discussions based on personal and comparative audit and feedback, we were able to tap into real-time communicated reflective processes and sketch a comprehensive image of the diversity of attributions and factors impacting participating GPs' motivation to change. Yet, the image could be confounded in three ways. The peer group setting, despite being beneficial to change motivation, might have induced participants to want to look their best. Besides, expressed motivation to change is no guarantee for actual change.(22, 36). Therefore, future work exploring the effects of audit and feedback in terms of patient outcomes and compliance with desired practice (cf. (6)) – both in the short and the long term – is crucial. Finally, the use of a specific type of audit and feedback with GP groups who share an interest in change management processes demands cautious interpretations in terms of transferability to other audit and feedback tools and other GP groups. Yet,

For peer review only

the current study's attributional perspective on audit and feedback has certainly enriched our understanding of the complexities of those processes that jointly foster improvement of GP professional practice: individual reflection and critical discussion.

For peer review only

Contributors

All authors contributed substantially to the conception, design, or execution of the reported study. MH and ISM, in collaboration with ND, took initiative for the study. ND, MV, MH and ISM designed the study and collected the data. JB participated in the interpretation of the data; MVB, ND and MV participated in data analysis. MVB was responsible for the write-up of the study. All authors critically revised its content and provided final approval of the version to be published.

Funding

This research received no specific grant from any funding agency in the public, commercial or not-for-profit sectors.

Competing interests

None declared.

Data sharing

The data for this paper may be obtained from the authors upon request.

For peer review only

References

1. Koefoed MM, Søndergaard J, Christensen Rd, et al. General practice variation in spirometry testing among patients receiving first-time prescriptions for medication targeting obstructive lung disease in Denmark: a population-based observational study. *BMC Fam Pract* 2013;14:113.

2. Millett C, Car J, Eldred D, et al. Diabetes prevalence, process of care and outcomes in relation to practice size, caseload and deprivation: national cross-sectional study in primary care. *J R Soc Med* 2007;100(6):275-83.

3. Nielen MMJ, Schellevis FG, Verheij RA. Inter-practice variation in diagnosing hypertension and diabetes mellitus: a cross-sectional study in general practice. *BMC Fam Pract* 2009;10:6.

4. Verstappen WM, van der Weijden T, Sijbrandij J, et al. Effect of a practice-based strategy on test ordering performance of primary care physicians: A randomized trial. *JAMA* 2003;289(18):2407-12.

5. Cervero RM, Gaines JK. The Impact of CME on Physician Performance and Patient Health Outcomes: An Updated Synthesis of Systematic Reviews. *J Contin Educ Health Prof* 2015;35(2):131-8.

6. Ivers N, Jamtvedt G, Flottorp S, et al. Audit and feedback: effects on professional practice and health care outcomes. *Cochrane Database Syst Rev* 2012(6):1-227.

7. O'Connell DL, Henry D, Tomlins R. Randomised Controlled Trial of Effect of Feedback on General Practitioners' Prescribing in Australia. *BMJ* 1999;318(7182):507-11.

8. Mugford M, Banfield P, O'Hanlon M. Effects Of Feedback Of Information On Clinical Practice: A Review. *BMJ* 1991;303(6799):398-402.

9. Axt-Adam P, van der Wouden JC, van der Does E. Influencing Behavior of Physicians Ordering Laboratory Tests: A Literature Study. *Med Care* 1993;31(9):784-94.

10. Verstappen WHJM, van der Weijden T, Dubois WI, et al. Improving Test Ordering in Primary Care: The Added Value of a Small-Group Quality Improvement Strategy Compared With Classic Feedback Only. *Ann Fam Med* 2004;2(6):569-75.

11. Brehaut JC, Colquhoun HL, Eva KW, et al. Practice feedback interventions: 15 suggestions for optimizing effectiveness. *Ann Intern Med* 2016;164(6):435-41.

For peer review only

12. Cooke LJ, Duncan D, Rivera L, et al. How do physicians behave when they participate in audit and feedback activities in a group with their peers? *Implement Sci* 2018;13(1):104.

13. Kluger AN, DeNisi A. The effects of feedback interventions on performance: A historical review, a meta-analysis, and a preliminary feedback intervention theory. *Psychol Bull* 1996;119(2):254.

14. Steele CM. The psychology of self-affirmation: Sustaining the integrity of the self. *Adv Exp Soc Psych* 1988:21:261-302.

15. Damschroder LJ, Aron DC, Keith RE, et al. Fostering implementation of health services research findings into practice: a consolidated framework for advancing implementation science. *Implement Sci* 2009;4(1):50.

16. Bandura A. Social learning theory. New Jersey: Prentice Hall; 1977.

17. Brown B. Harnessing opportunities for quality improivement from primary care electronic health records [PhD dissertation]: University of Manchester; 2018.

18. Kusurkar RA, Artino AR, Ten Cate TJ. Motivation and learning. In: Bhuiyan PS, Rege NN, Supe A, editors. The art of teaching medical students. New Delhi: Reed Elsevier India Pvt. Ltd. 2015:62-76.

19. Fox R, Mazmanian P, Putnam RW. Changing and learning in the lives of physicians. New York: Praeger; 1989.

20. Grol R, Wensing M. What drives change? Barriers to and incentives for achieving evidence-based practice. *Med J Aus* 2004;180(6 Suppl):S57-60.

21. Kanouse DE, Jacoby I. When does information change practitioners' behavior? *Int J Technol Assess Health Care* 1988;4(1):27-33.

22. Wakefield J, Herbert CP, Maclure M, et al. Commitment to change statements can predict actual change in practice. *J Contin Educ Health Prof* 2003;23(2):81-92.

23. Palmer RH, Louis TA, Hsu L-N, et al. A Randomized Controlled Trial of Quality Assurance in Sixteen Ambulatory Care Practices. *Med Care* 1985;23(6):751-70.

24. Foy R, MacLennan G, Grimshaw J, et al. Attributes of clinical recommendations that influence change in practice following audit and feedback. *J Clin Epidemiol* 2002;55(7):717-22.

25. Cook DA, Artino AR. Motivation to learn: an overview of contemporary theories. *Med Educ* 2016;50(10):997-1014.

For peer review only

26. Weiner B. An Attributional Theory of Achievement Motivation and Emotion. *Psychol Rev* 1985;92(4):548-73.

27. Weiner B. Intrapersonal and Interpersonal Theories of Motivation from an Attributional Perspective. *Educ Psychol Rev* 2000;12(1):1-14.

28. Vektis. Zorgprisma Publiek. Huisartsenzorg. 2017. https://www.zorgprismapubliek.nl/informatie-over/huisartsenzorg/huisartsenzorg/ (accessed June 2017).

29. Krueger RA, Casey MA. Focus groups. A practical guide for applied research. Thousand Oaks, CA: SAGE Publications, Inc. 2000.

30. Tong A, Sainsbury P, Craig J. Consolidated criteria for reporting qualitative research (COREQ): a 32-item checklist for interviews and focus groups. *Int J Qual Health Care* 2007;19(6):349-57.

31. Braun V, Clarke V. Using thematic analysis in psychology. *Qual Res Psychol* 2006;3(2):77-101.

32. Trietsch J, van der Weijden T, Verstappen W, et al. A cluster randomized controlled trial aimed at implementation of local quality improvement collaboratives to improve prescribing and test ordering performance of general practitioners: Study Protocol. *Implement Sci* 2009;4:6.

33. Sargeant J, Lockyer J, Mann K, et al. Facilitated reflective performance feedback: developing an evidence-and theory-based model that builds relationship, explores reactions and content, and coaches for performance change (R2C2). *Acad Med* 2015;90(12):1698-706.

34. Carless D, Salter D, Yang M, et al. Developing sustainable feedback practices. *Stud High Educ* 2011;36(4):395-407.

35. Cooke LJ, Duncan D, Rivera L, et al. The Calgary Audit and Feedback Framework: a practical, evidence-informed approach for the design and implementation of socially constructed learning interventions using audit and group feedback. *Implement Sci* 2018;13(1):136.

36. Overton GK, McCalister P, Kelly D, et al. Practice-based Small Group Learning: How health professionals view their intention to change and the process of implementing change in practice. *Med Teach* 2009;31(11):e514-e20.

For peer review only

BMJ Open

What motivates General Practitioners to change practice behavior? A qualitative study of audit and feedback group sessions in Dutch General Practice

Journal:	*BMJ Open*
Manuscript ID	bmjopen-2018-025286.R3
Article Type:	Research
Date Submitted by the Author:	27-Mar-2019
Complete List of Authors:	van Braak, Marije; Erasmus Medical Centre, General Practice<br>Visser, Mechteld; Amsterdam University Medical Centre, General Practice<br>Holtrop, Marije; General Practitioners Holtrop and Sieben<br>Statius Muller, Ilona; Ubbens and Statius Muller General Practitioners<br>Bont, Jettie; Amsterdam University Medical Centre, General Practice<br>van Dijk, Nynke; Amsterdam University Medical Centre, General Practice
**Primary Subject Heading**:	General practice / Family practice
Secondary Subject Heading:	Medical education and training, Qualitative research
Keywords:	MEDICAL EDUCATION & TRAINING, PRIMARY CARE, QUALITATIVE RESEARCH

SCHOLARONE™
Manuscripts

What motivates General Practitioners to change practice behavior? A qualitative study of audit and feedback group sessions in Dutch General Practice

Correspondence:

Marije van Braak, MSc

Department of General Practice, Erasmus Medical Centre

Postbus 2040, Dr. Molewaterplein 40, 3015 GD Rotterdam, the Netherlands

m.vanbraak@erasmusmc.nl

+31 10 7042518

Mechteld R. M. Visser, PhD

Department of General Practice, Amsterdam University Medical Centre, Amsterdam, the Netherlands

Marije Holtrop, MD, GP

General Practitioners Holtrop and Sieben

Amsterdam, the Netherlands

Ilona Statius Muller, MD, GP

Ubbens and Statius Muller General Practitioners

Amsterdam, the Netherlands

Jettie Bont, MD, PhD, GP

Department of General Practice, Amsterdam University Medical Centre, Amsterdam, the Netherlands

Nynke van Dijk, Professor, MD, PhD

Department of General Practice, Amsterdam University Medical Centre, Amsterdam, the Netherlands

Word count: 4067

For peer review only

ABSTRACT

Objectives Adopting an attributional perspective, the current article investigates how audit and feedback group sessions contribute to GPs' motivation to change their practice behavior to improve care. We focus on the contributions of the audit and feedback itself (content) and the group discussion (process).

Methods Four focus groups, comprising a total of 39 participating Dutch GPs, discussed and compared audit and feedback of their practices. The focus groups were analyzed thematically.

Results Audit and feedback contributed to GPs' motivation to change in two ways: by raising awareness about aspects of their current care practice and by providing indications of the possible impact of change. For these contributions to play out, the audit and feedback should be reliable and valid, specific, recent and recurrent, and concern GPs' own practices or practices within their own influence sphere. Care behavior attributed to external, uncontrollable, or unstable causes would not induce change. The added value of the group is twofold as well: group discussion contributed to GPs' motivation to change by providing a frame of reference and by affording insights that participants would not have been able to achieve on their own.

Conclusions In audit and feedback group sessions, both audit and feedback information and group discussion can valuably contribute to GPs' motivation to change care practice behavior. Peer interaction can positively contribute to explore alternative practices and avenues for improvement. Local or regional peer meetings would be beneficial in facilitating reflection and discussion. An important avenue for future studies is to explore the contribution of audit and feedback and small-group discussion to *actual* practice change.

For peer review only

STRENGTHS AND LIMITATIONS OF THIS STUDY

- Framed within Attribution Theory, the study provides a novel perspective on audit and feedback.
- Focus group discussions on personal and comparative audit and feedback allowed us to tap into real-time reflective processes.
- Qualitative analysis of recorded interaction between GPs allowed for detailed insight into the value of peer interaction in discussions on practice change.
- Voluntary GP participation may have resulted in a sample of participants with a special interest in audit and feedback and behavior change.
- The study focused only on effects of audit and feedback and group discussion on intended, not actual, practice change.

For peer review only

INTRODUCTION

In taking the Hippocratic oath, general practitioners – and other doctors as well – express their intention to treat patients to the best of their ability. Yet, care practices of general practitioners show substantial unintended variation.(1-3) Part of this diversity is induced by external practice or population factors, such as practice size.(2) Individual factors also play a role: general practitioners' knowledge, skills, experience, interests and preferences can induce between-practice variation. This variation might be related to differences in clinical judgment based on considerations of evidence, clinical experience and patient preferences,(4) but sometimes is not intended and may lead to lower patient care quality.

For general practitioners (GPs) to develop their professional practice, they should be aware of this unintended variation. Reflection on between-practice variation can lead to adjustments in professional care behavior, eventually improving the quality of patient care.(5) Audit and feedback on GPs' performance can effectively improve professional practice, albeit under certain optimally-designed conditions and in the right context.(6) Several factors influencing the effectiveness of feedback have been researched. These include the level of feedback detail,(7) its timing, (8, 9) and the interactivity of the feedback-giving process.(4, 5, 9, 10) Based on expert interviews, systematic reviews, and experience, Brehaut and colleagues suggest that practice feedback interventions can be optimized by, amongst others, linking it to established goals, providing feedback in more than one way, minimizing extraneous cognitive load for feedback recipients, increasing the credibility of the data and preventing defensive reactions to feedback.(11) Additionally, recent research on group audit and feedback points at the added value of socially constructed learning activities in audit and feedback group sessions.(12)

The theoretical framing of audit and feedback research is diverse, ranging from feedback theories (e.g., Feedback Intervention Theory, (13)) to psychological theories (e.g. Self Affirmation Theory, (14)) to implementation theories (e.g. Consolidated framework for implementation research, (15)) to learning theories (e.g. Social Learning Theory, (16)).(17) In our study, we explicitly focus on GPs' *motivation* to change. This perspective on audit and feedback has not been used before. Yet, motivation is essential to learning and change processes(18) and has been found to influence change

For peer review only

behavior of various physicians.(19-21) In a study on physicians' prescription behavior, Wakefield et al.(22) found that physicians who expressed a commitment to change following participation in a continuing medical education program using interactive small groups were significantly more likely to change their targeted prescription practices in the following half year. Contradictory evidence, however, has also been reported.(23, 24) Palmer(23) assessed the motivation of health professionals to change their practices and found that they improved on tasks for which they reported to have *limited* motivation. This counterintuitive finding can be explained by the significant *system* changes (in addition to individual changes) required to change practices for which professionals' motivation was high. Thus, the relation between motivation and care practice improvement may be less straightforward than originally anticipated.

The reported variability in the effect of motivation on actual care practice change can be explained by attributional processes that impact an individual's motivation to change.(25) For example, during audit and feedback meetings GPs might attribute practice variation to 'the system', particular patients, or fellow healthcare professionals (i.e., hold others responsible for practice variation). In such a case, the GPs are not likely to be inclined to change their own professional practice behavior (cf. (11)). Thus, GPs' attributions are significant in processes of general practice change and need careful consideration in the context of audit and feedback. In our study, therefore, we adopted an attributional perspective on audit and feedback.

In the Dutch context, audit and feedback have become increasingly important for GP professional development. Historically, GPs use pharmacological feedback from pharmacists and their electronic health records to improve prescribing behavior – mainly in educational group sessions. In the last decade, GPs have started to use these sessions to discuss diagnostic procedures. At the same time, insurance companies have started to request from GPs information on quality indicators. As GPs expressed a need for audit and feedback sessions based on these quality indicators, the Dutch movement 'Optimale zorg-Dappere dokters' [Optimal care-Daring doctors] initiated an audit and feedback group intervention, aiming to encourage GPs' self-reflection and optimize care.

Group-based critical self-evaluation based on quality indicators is not standard in contemporary quality policies of the medical profession, but can be beneficial to stimulate self-

For peer review only

reflection for optimal care.(12) Understanding how participants respond to the audit and feedback data will facilitate improvement of interventions. To this end, we qualitatively investigated how audit and feedback group sessions contribute to GPs' motivation to change their practice behavior to improve care. We focused on GPs' attributions regarding both content and process of the audit and feedback group sessions by asking:

1. How does the audit and feedback itself contribute to GPs' motivation to change?
2. How does the group discussion contribute to GPs' motivation to change?

THEORETICAL FRAMEWORK

The role of attributions in motivation and behavior change is described in Weiner's Attribution Theory.(26, 27) This theory indicates that humans "have a tacit goal of understanding and mastering themselves and their environment" and "establish cause-effect relationships for events in their lives".(25) Occurring events lead to attribution, a process of (often subconsciously) seeking an explanation by hypothesizing perceived personal and environmental causes (e.g., ability, effort, luck, task difficulty, mood, health, other people, etc.). These causes can be organized along three causal dimensions: locus (i.e., internal or external to the individual), stability (i.e., stable, fixed or unstable, likely to change), and controllability (i.e., within or outside the individual's control). Based on the specific combination of values in each dimension, occurring events are interpreted as psychologically meaningful responses. As such, the individual's *interpretation* of a particular event determines their response to that event. Typically, bad luck is interpreted as external, unstable and uncontrollable – invoking no or possibly only a passive reaction from the individual; personal effort is internal, changeable, and controllable – and can thus be influenced by an individual's actions; and innate skill is internal, largely fixed, and uncontrollable – making it an unlikely subject of change.(25)

METHODS

Ethics

The Medical Ethics Review Committee of the Amsterdam Academic Medical Centre confirmed that the Medical Research Involving Human Subjects Act does not apply to this study and that an official approval of this study by the committee was not required (reference number W18_200 # 18.241).

Patient and Public Involvement

For peer review only

No patient or public were involved. The development of the research question was informed by two practicing GPs (MH, ISM), who also participated in the design of the study. The results will be disseminated to participants via an email informing GPs about the main results and focusing on issues of future audit and feedback implementation.

Setting

The study was carried out in the region of Amsterdam. Dutch general practice plays an important role in the Dutch health care system, since specialist care in the Netherlands is only accessible upon referral by a GP.(28) All Dutch citizens are registered at a GP-practice in their regional area. Patients visit their GP when faced with a medical problem (except in life-threatening situations). The GP collects and evaluates all relevant medical information. Consequent treatment decisions and referrals to a medical specialist are taken together with the patient. Costs of care are covered by healthcare insurances, which are compulsory for people who live or work in the Netherlands and which include at least basic health care.

Audit and feedback information is gathered and provided to GPs by Vektis, a Dutch center for information and standardization of health insurance. This information is based on declarations of health care costs to insurance companies. It provides data on indicators regarding practice population, consultations, interventions, prescription, and referral rates compared with a standardized Dutch practice, corrected for age, gender, social-economic status and disease severity of the population.

Data collection

We conducted focus group discussions(29, 30) with GPs of four regional GP groups within the Amsterdam region. GP groups interested in this audit and feedback intervention could participate if their practices were in the Amsterdam region. General practitioners were approached and informed about the research purpose and participation practicalities via an information letter. Participating GPs signed informed consent prior to the discussions and sent the audit and feedback information that their practices received from Vektis (data from 2012-2014) to the research team for analysis. The focus group discussions were held between June 2016 and March 2017 at a participating GP's practice, lasted approximately 1.5 hours and were audio-taped for transcription purposes.

For peer review only

Focus group discussions were facilitated by a moderator (ND or MV). They guided the group through the data reports. Resembling Cooke et al.'s intervention(12), an aggregate comparative report of quality indicators (selected for their relevance by each focus group in a session preceding the audit and feedback session) was projected for everyone to see; actual practice information was only available to GPs of that practice, but was shared with other GPs if relevant to the discussion or upon practitioners' own initiation. The facilitator encouraged participants to ask questions or share remarkable or unexpected aspects of their individual feedback information, facilitated interpretation of the feedback data (e.g., by explaining how it is constructed), and probed participants to discuss the consequences of the data for their future daily practice. Each focus group discussion concluded with an exploration of potential issues relevant for follow-up sessions (not reported on in this paper).

The four focus group interactions were transcribed verbatim and anonymized by deleting geographical and personal names. Fragments unintelligible due to simultaneous speech or laughing were transcribed as [unintelligible] and included a timestamp to facilitate fragment location at a later moment of analysis if needed.

Analysis

Transcripts were analyzed using theoretical thematic analysis (31); audiotapes were consulted to improve interpretation where necessary. Key concepts derived from Attribution Theory, e.g. 'external attribution', formed the initial framework for data coding. MB first coded one transcript, supplementing the initial codes with codes derived inductively from the data (e.g., *gaining information* or *evaluation*). MB and ND discussed the coding of this first transcript to ensure coding reliability. Applying constant comparison, several codes were modified or merged for code reduction. Next, MB and ND independently coded a second transcript, adding additional open codes where needed. They discussed their codings in detail until agreement was reached. MB modified the coding of the first two transcripts accordingly and used the final coding scheme to code the two remaining transcripts. Again, MB and ND discussed fragments that could not unambiguously be coded until agreement was reached. Although the content of the last focus groups only partly resembled the content of the first two focus groups, the existing codes sufficed to cover the content. This provided evidence for data saturation. As

For peer review only

a final step, MB organized the code themes into a coherent and internally consistent account of what motivates GPs to change.

Reflexivity

Four members of the research team were medical doctors (MH, ISM, JB, ND), three of whom were practicing GPs (MH, ISM, JB). The practicing GPs approached ND to initiate the study, induced by their practice experience. They did not participate in data collection or analysis to prevent interaction between their individual experience and the data collection process. One of the focus group facilitators was a medical doctor (not practicing). Analysis was primarily done by MB, who has no medical training and therefor was most distant to the content discussed. This benefitted a broad outlook on the data.

RESULTS

Focus groups were attended by 39 GPs from four regional groups (7-10 GPs per focus group). Participants of three focus groups all had practices in an urban area; practices of the fourth focus group's participants were situated in a rural area. GPs' age ranged from 30 to 65 years (the majority being over 50 years of age). Approximately 75 percent of participating GPs work in a practice together with a partner, with the remainder working alone or in a group practice.

Generally, discussions of audit and feedback items commenced with resolving potential interpretation difficulties. Subsequently, GPs construed an understanding of the information, focusing on probable explanations for deviations from average or between practices. These tended to be followed by GPs expressing their motivations to change.

In the following, we first present aspects of the audit and feedback that contributed to GPs' motivation to change behavior. We specifically focus on the attributions that GPs used to explain their hesitation to change. Next, we present aspects of the group discussion that contributed to GPs' motivation to change.

Motivation to change: contribution of audit and feedback information

Two important contributions of audit and feedback information to GPs' motivation to change emerged from our analysis of the focus group discussions. First, the audit and feedback motivated GPs to change *by raising awareness about aspects of their current care practice*. A heightened awareness

For peer review only

of one’s current practices as reported in the audit and feedback, could lead to the realization that actual care practices differed extensively from perceived care practices or from the norm. This insight frequently induced GPs to express intentions to further reflect on or take additional steps towards practice change, an example of which is presented in Box 1.

Box 1 *Focus group O*

1	GP A	Well, what I do want to do is check all our cyriax cases [i.e., orthopaedic corticosteroid
2		injections] to see what the indications were. That is quite a job and it would be very nice if we
3		could receive that audit and feedback. But this finding has already provoked me to look that up
4		in my own Electronic Health Record to see how many have been done.
5	CHA	Yes.
6	GP A	Then we would really have something to talk about, I think.

For peer review only

As this excerpt reveals, being confronted with specific audit and feedback about one’s own practice (e.g., the finding that the number of cyriax cases, for example a corticosteroid knee injection, deviates considerably from the norm, line 3) can lead to undertaking specific steps to understand and potentially adapt one’s care practices.

Second, the audit and feedback further contributes to GPs’ motivation to change *by providing insight into the degree of deviation from norms*. If deviations from ‘average’ practice are large, changing practice would have a large impact. Minimal deviations from the norm, on the contrary, are judged irrelevant to future practice change. Similarly, the number of patients that are included in a figure signal the impact of potential change. Deviations – either negative or positive – in practice behavior were less likely a driver for change if only a few patients were involved (Box 2).

Box 2 *Focus group P*

1	GP A	There is one other thing that I appreciate about these figures, sometimes deviations are
2		enormous but then it’s only about 10 patients, one isn’t going to change policy on that.

For these two contributions of audit and feedback to play out, however, GPs pointed out that several conditions have to be met. First, the audit and feedback information *should be reliable and valid*. Suspicion of unreliability of the audit and feedback induces insecurity about possible future actions; one GP said: “it doesn’t match with how I feel about it (…), so I don’t really know what I should or could do with that”. According to another GP, only reliable figures that resemble the GP’s own behavior can rightfully trigger change. Besides being reliable, figures should also be valid.

For peer review only - http://bmjopen.bmj.com/site/about/guidelines.xhtml

Examples of information that GPs considered to be invalid are: prescriptions that were recorded as prescribed by the GP, but were in fact specialist prescriptions, figures that simply *could not be true* (e.g. only three recorded prescriptions of a medicine that is very commonly prescribed), or drastic changes in particular prescription behavior from one year to another, while prescription policies were unchanged. If the reader cannot tell what comprises the figures, it remains unclear why increases and decreases in prescriptions, referrals, and treatments occur and to whom (or what) these changes can be attributed. As GP A points out in Box 3, the data's construction is key to its interpretation.

Box 3 *Focus group V*

1	GP A	But then one soon asks oneself: "How did they get to these figures? How is all of this calculated,
2		if the difference is so large, we haven't started working completely differently a year later". So
3		there is something there. That does give a lot of interesting information (…). It always comes
4		down to: What are your norms, why - how are things actually counted? Yes, that's when things
5		get terribly difficult. That says a lot about the reliability.
6	GP B	I would say they have started to count in a different way.

Perceived limited reliability and clarity frequently induced external attributions, i.e. explanations of feedback information by causes external to the GPs influence sphere. If attributed externally, no change talk would follow. As such, unreliability and invalidity of the data compromise the potential contribution of the audit and feedback information to GPs motivation to change care practices.

Second, the audit and feedback information *should be specific.* GPs' motivation to change would benefit from broad themes being split up into smaller subthemes (e.g., ECGs for specific problems instead of one figure for all ECGs made). The contribution of audit and feedback to GPs' motivation to change would be increased if specific prescriptions or patient information would be available on request. This would help understand, for example, extreme prescription rates or costs (as one GP with high costs wondered: "Is that because of that Augmentin for that cat bite?"). Having the option to link prescriptions, referrals, and costs to specific patients would point GPs at potential behavior for change.

Third, audit and feedback information *should be recent and recurrent*. Short term feedback is agreed upon as being critical to the effectiveness of audit and feedback as facilitator of change. GPs do not feel the urge to 'learn' from figures that represent their behavior registered three or more years ago. The feedback should not only be recent, however, but also be recurrent, as Box 4 shows.

For peer review only

Box 4 *Focus group P*

1	GP A	Those figures are very broad and big, really, so I find it difficult to - if one gets back something
2		small from your figures, something that you can improve on easily and if one gets back the
3		figures again after half a year, then I would be more likely to show behavior change.

Fourth, the audit and feedback *should concern GPs' own practices or practices within their own influence sphere*. An example of care practices *outside* the GPs' control are specialist prescriptions. Some GPs suggested to talk to a specialist to discuss deviant figures or refuse particular referrals. More commonly, however, such figures are unlikely subjects for change. GPs would attribute the deviations from 'average' practice represented by these figures to external sources. Examples of such sources are non-GP health professionals (in case of in-hospital treatment) a GP-in-training (whose presence could result in more applications for radiology or lab diagnostics), the practice location (which, for example, might result in fewer home visits if located close to an old-age home or in the care center), regulations (e.g, codes of conduct), and time issues (see Box 5).

Box 5 *Focus group OD*

1	GP A	I do tell them quite often to come - make a new appointment, but I do have too few consults
2		already as well.
3	GP B	Oh so that doesn't help either.
4	GP A	So that doesn't help either.
5	GP C	We cannot even schedule more consults.
6	GP D	Full is full.
7	GP C	Yes, only if you want to continue working through the evening.

Despite being potentially problematic, audit and feedback elements that pertain to issues far beyond GPs' control (i.e., external attribution) do not induce motivation to change their own practices.

Motivation to change: contribution of group discussion

In the current study's audit and feedback sessions, the group contributed to GPs' motivation to change in two ways. First, and most importantly, the presence of peers *provided a frame of reference* for interpretation and evaluation of feedback figures. During the focus group discussions, GPs could compare their feedback figures. Comparison can be very informative, as one GP points out in Box 6.

Box 6 *Focus group OD*

1	GP A	I think that is interesting, when we see - when I see that I deviate from the national average, and
2		we all deviate, then you think: what do we do about that?
3	GP B	Sure.

For peer review only

4	GP A	If only mine deviates, then I think: well, I have to do something about that.

Apparently, the need (and motivation) to change practices is more pressing if only one GP's practice deviates from the norm compared to deviation common to all participating GPs. Common deviations are often attributed to demographical or geographical characteristics. Comparing each other's audit and feedback thus functions as a filter, isolating idiosyncratic practice-related variation from region-bound practice variation.

Second, the contribution of group members can yield important *insights that participants would not have been able to achieve on their own*. At times, discussing care practices led to an explicitly formulated realization that particular practices had to be adjusted for reasons proposed during the discussion. In the excerpt displayed in Box 7, for instance, one participating GP realized that his hesitation to plan double consults (20 minutes for patients with multiple complaints) instead of the common 10-minute consult (the standard option in Dutch general practice) was unjustified.

Box 7 *Focus group O*

1	GP A	This is such an eye opener for me, you know? I constantly feel guilty when I plan twenty-
2		minute consults twice a day, because then my assistants won't be able to schedule
3		enough patients – that's what I think. But I can only do that!
4	GP B	But darling, we are manning just slightly more patients with twice as many practitioners as you
5		have.
6	GP A	Yes, that's true.
7	GP B	You know, I mean - that is how it is, really.
8	GP C	Yes, so you need an extra doctor, and more double consults.
9	GP A	And a room.
10	GP C	Yes, and if we charge for the double consults as well, then an extra doctor does not cost a thing.
11	GP D	Indeed, then you recover the expenses easily.

Other GPs participating in this interaction are used to planning double consults and do not experience the time issues that GP A mentions in lines 1-2. The reason behind the differences in the number of double consults planned, GP B poses, is probably understaffing (line 4). Following this suggestion, GP C and GP D formulate an explicit need for change (lines 5-6), and potential objections to the proposed change are warded off in lines 7-8. This excerpt shows the benefits of discussing one's audit and feedback with fellow GPs: peers can point out problematic issues or solutions that one has not considered themselves. Also, peers can encourage each other to explore a solution and motivate each other to change – as we see happens in Box 7.

For peer review only

Notwithstanding their contributions to GPs' motivation to change, however, group discussions sometimes merely initiated sharing of motivations behind and reflections on practice behavior without triggering change talk. This type of motivated sharing of best practices occurred quite frequently throughout the group discussions. In general, though, group discussion is perceived to facilitate interpretation of audit and feedback and evaluations of the need for practice change.

DISCUSSION

The present study qualitatively investigated how audit and feedback group sessions can contribute to GPs' motivation to change practice behavior to improve care. We framed GPs' responses to the group audit and feedback sessions with Attribution Theory. This theory contends that an individual's motivation to change behavior is contingent on their interpretation of the cause behind that behavior, i.e. whether the cause is internal or external, is stable, and is controllable, is central to this approach. Understanding GPs' attributions of behavior presented during audit and feedback is therefore essential for designing interventions aimed at changing suboptimal care practices.

The presented analysis shows that audit and feedback information can contribute to motivation to change by raising awareness about current practice. At times, that awareness propels GPs to the next step towards change.(20) Audit and feedback can also contribute to motivation to change by providing an indication of the potential impact of change in terms of degree of deviation and number of patients, prescriptions, etc. involved. Generally, the lower the impact of change, the lower GPs' motivation to change. A pragmatic consideration seems to be at play here.(23) Even if GPs interpret the behavior as controllable (something can be done about it), stable (it does not occur randomly), and within their own action range (internal locus), the effort does not outweigh the benefit of change. In these cases, GPs' attributions would not explain the contribution of audit and feedback to GPs' motivation to change. In general, though, the extent to which the audit and feedback pertain to GPs' *individual, controllable, and changeable* behavior is a strong factor in inducing GPs' expressions of change intention – in line with the tenet of Attribution Theory.(25)

Our findings also point to the key role of *collectively discussing* audit and feedback. As indicated by Trietsch et al., social influence and norms affect participants' reflective behavior and corresponding intentions to change current practice during peer interaction.(32) Whereas Ivers et al.

For peer review only

conclude that there is very limited evidence for peer-comparison audit and feedback being either more or less effective than individual performance information,(6) our data show that peer comparison in general and group processes in particular stimulate critical appraisal of the audit and feedback and the need for behavioral change.(12, 33-35) Peer comparison provides an interpretative framework for individual practice data and peer interactivity provides ample opportunities to explore alternative practices and promising avenues for improvement.(5, 10)

Practically, our findings indicate that significant adjustment of contemporary Dutch audit and feedback practices is required to assure prompt and profitable use for improvement of professional practice. To potentially effectuate change, audit and feedback ought to be *reliable*, *valid, specific*, *recent* and *recurrent* (cf. recommendations in similar and other research contexts(7-9)(11)). We suggest to employ already accessible practice-related information (e.g., in the Electronic Health Record) as a starting point for an informative, easily accessible, and adaptively employable application serving improvement of GPs' professional practice. Additionally, frequent meetings with GPs practicing in the same local or regional area beyond the one currently investigated would be very valuable to facilitate group reflection and discussion across the country. This would promote self-governance of the Dutch GPs, in appreciation of the common needs that led to the Optimal Care-Daring Doctors movement that started this audit and feedback initiative. Future evaluation research on such interventions would be essential to ensure progressive refinement of the intervention.

By conducting focus group discussions based on personal and comparative audit and feedback, we were able to tap into real-time communicated reflective processes and sketch a comprehensive image of the diversity of attributions and factors impacting participating GPs' motivation to change. Yet, the image could be confounded in three ways. The peer group setting, despite being beneficial to change motivation, might have induced participants to want to look their best. Besides, expressed motivation to change is no guarantee for actual change.(22, 36). Therefore, future work exploring the effects of audit and feedback in terms of patient outcomes and compliance with desired practice (cf. (6)) – both in the short and the long term – is crucial. Finally, the use of a specific type of audit and feedback with GP groups who share an interest in change management processes demands cautious interpretations in terms of transferability to other audit and feedback tools and other GP groups. Yet,

For peer review only

the current study's attributional perspective on audit and feedback has certainly enriched our understanding of the complexities of those processes that jointly foster improvement of GP professional practice: individual reflection and critical discussion.

For peer review only

Contributors

All authors contributed substantially to the conception, design, or execution of the reported study. MH and ISM, in collaboration with ND, took initiative for the study. ND, MV, MH and ISM designed the study and collected the data. JB participated in the interpretation of the data; MVB, ND and MV participated in data analysis. MVB was responsible for the write-up of the study. All authors critically revised its content and provided final approval of the version to be published.

Funding

This research received no specific grant from any funding agency in the public, commercial or not-for-profit sectors.

Competing interests

None declared.

Data sharing

The data for this paper may be obtained from the authors upon request.

For peer review only

References

1. Koefoed MM, Søndergaard J, Christensen Rd, et al. General practice variation in spirometry testing among patients receiving first-time prescriptions for medication targeting obstructive lung disease in Denmark: a population-based observational study. *BMC Fam Pract* 2013;14:113.

2. Millett C, Car J, Eldred D, et al. Diabetes prevalence, process of care and outcomes in relation to practice size, caseload and deprivation: national cross-sectional study in primary care. *J R Soc Med* 2007;100(6):275-83.

3. Nielen MMJ, Schellevis FG, Verheij RA. Inter-practice variation in diagnosing hypertension and diabetes mellitus: a cross-sectional study in general practice. *BMC Fam Pract* 2009;10:6.

4. Verstappen WM, van der Weijden T, Sijbrandij J, et al. Effect of a practice-based strategy on test ordering performance of primary care physicians: A randomized trial. *JAMA* 2003;289(18):2407-12.

5. Cervero RM, Gaines JK. The Impact of CME on Physician Performance and Patient Health Outcomes: An Updated Synthesis of Systematic Reviews. *J Contin Educ Health Prof* 2015;35(2):131-8.

6. Ivers N, Jamtvedt G, Flottorp S, et al. Audit and feedback: effects on professional practice and health care outcomes. *Cochrane Database Syst Rev* 2012(6):1-227.

7. O'Connell DL, Henry D, Tomlins R. Randomised Controlled Trial of Effect of Feedback on General Practitioners' Prescribing in Australia. *BMJ* 1999;318(7182):507-11.

8. Mugford M, Banfield P, O'Hanlon M. Effects Of Feedback Of Information On Clinical Practice: A Review. *BMJ* 1991;303(6799):398-402.

9. Axt-Adam P, van der Wouden JC, van der Does E. Influencing Behavior of Physicians Ordering Laboratory Tests: A Literature Study. *Med Care* 1993;31(9):784-94.

10. Verstappen WHJM, van der Weijden T, Dubois WI, et al. Improving Test Ordering in Primary Care: The Added Value of a Small-Group Quality Improvement Strategy Compared With Classic Feedback Only. *Ann Fam Med* 2004;2(6):569-75.

11. Brehaut JC, Colquhoun HL, Eva KW, et al. Practice feedback interventions: 15 suggestions for optimizing effectiveness. *Ann Intern Med* 2016;164(6):435-41.

For peer review only

12. Cooke LJ, Duncan D, Rivera L, et al. How do physicians behave when they participate in audit and feedback activities in a group with their peers? *Implement Sci* 2018;13(1):104.

13. Kluger AN, DeNisi A. The effects of feedback interventions on performance: A historical review, a meta-analysis, and a preliminary feedback intervention theory. *Psychol Bull* 1996;119(2):254.

14. Steele CM. The psychology of self-affirmation: Sustaining the integrity of the self. *Adv Exp Soc Psych* 1988:21:261-302.

15. Damschroder LJ, Aron DC, Keith RE, et al. Fostering implementation of health services research findings into practice: a consolidated framework for advancing implementation science. *Implement Sci* 2009;4(1):50.

16. Bandura A. Social learning theory. New Jersey: Prentice Hall; 1977.

17. Brown B. Harnessing opportunities for quality improivement from primary care electronic health records [PhD dissertation]: University of Manchester; 2018.

18. Kusurkar RA, Artino AR, Ten Cate TJ. Motivation and learning. In: Bhuiyan PS, Rege NN, Supe A, editors. The art of teaching medical students. New Delhi: Reed Elsevier India Pvt. Ltd. 2015:62-76.

19. Fox R, Mazmanian P, Putnam RW. Changing and learning in the lives of physicians. New York: Praeger; 1989.

20. Grol R, Wensing M. What drives change? Barriers to and incentives for achieving evidence-based practice. *Med J Aus* 2004;180(6 Suppl):S57-60.

21. Kanouse DE, Jacoby I. When does information change practitioners' behavior? *Int J Technol Assess Health Care* 1988;4(1):27-33.

22. Wakefield J, Herbert CP, Maclure M, et al. Commitment to change statements can predict actual change in practice. *J Contin Educ Health Prof* 2003;23(2):81-92.

23. Palmer RH, Louis TA, Hsu L-N, et al. A Randomized Controlled Trial of Quality Assurance in Sixteen Ambulatory Care Practices. *Med Care* 1985;23(6):751-70.

24. Foy R, MacLennan G, Grimshaw J, et al. Attributes of clinical recommendations that influence change in practice following audit and feedback. *J Clin Epidemiol* 2002;55(7):717-22.

25. Cook DA, Artino AR. Motivation to learn: an overview of contemporary theories. *Med Educ* 2016;50(10):997-1014.

For peer review only

26. Weiner B. An Attributional Theory of Achievement Motivation and Emotion. *Psychol Rev* 1985;92(4):548-73.

27. Weiner B. Intrapersonal and Interpersonal Theories of Motivation from an Attributional Perspective. *Educ Psychol Rev* 2000;12(1):1-14.

28. Vektis. Zorgprisma Publiek. Huisartsenzorg. 2017. https://www.zorgprismapubliek.nl/informatie-over/huisartsenzorg/huisartsenzorg/ (accessed June 2017).

29. Krueger RA, Casey MA. Focus groups. A practical guide for applied research. Thousand Oaks, CA: SAGE Publications, Inc. 2000.

30. Tong A, Sainsbury P, Craig J. Consolidated criteria for reporting qualitative research (COREQ): a 32-item checklist for interviews and focus groups. *Int J Qual Health Care* 2007;19(6):349-57.

31. Braun V, Clarke V. Using thematic analysis in psychology. *Qual Res Psychol* 2006;3(2):77-101.

32. Trietsch J, van der Weijden T, Verstappen W, et al. A cluster randomized controlled trial aimed at implementation of local quality improvement collaboratives to improve prescribing and test ordering performance of general practitioners: Study Protocol. *Implement Sci* 2009;4:6.

33. Sargeant J, Lockyer J, Mann K, et al. Facilitated reflective performance feedback: developing an evidence-and theory-based model that builds relationship, explores reactions and content, and coaches for performance change (R2C2). *Acad Med* 2015;90(12):1698-706.

34. Carless D, Salter D, Yang M, et al. Developing sustainable feedback practices. *Stud High Educ* 2011;36(4):395-407.

35. Cooke LJ, Duncan D, Rivera L, et al. The Calgary Audit and Feedback Framework: a practical, evidence-informed approach for the design and implementation of socially constructed learning interventions using audit and group feedback. *Implement Sci* 2018;13(1):136.

36. Overton GK, McCalister P, Kelly D, et al. Practice-based Small Group Learning: How health professionals view their intention to change and the process of implementing change in practice. *Med Teach* 2009;31(11):e514-e20.

For peer review only
